# ^1^H HR-MAS NMR Based Metabolic Profiling of Cells in Response to Treatment with a Hexacationic Ruthenium Metallaprism as Potential Anticancer Drug

**DOI:** 10.1371/journal.pone.0128478

**Published:** 2015-05-29

**Authors:** Martina Vermathen, Lydia E. H. Paul, Gaëlle Diserens, Peter Vermathen, Julien Furrer

**Affiliations:** 1 Department of Chemistry and Biochemistry, University of Bern, Bern, Switzerland; 2 Departments of Clinical Research and Radiology (AMSM), University of Bern, Bern, Switzerland; Instituto de Investigación Sanitaria INCLIVA, SPAIN

## Abstract

^1^H high resolution magic angle spinning (HR-MAS) NMR spectroscopy was applied in combination with multivariate statistical analyses to study the metabolic response of whole cells to the treatment with a hexacationic ruthenium metallaprism [**1**]^6+^ as potential anticancer drug. Human ovarian cancer cells (A2780), the corresponding cisplatin resistant cells (A2780cisR), and human embryonic kidney cells (HEK-293) were each incubated for 24 h and 72 h with [**1**]^6+^ and compared to untreated cells. Different responses were obtained depending on the cell type and incubation time. Most pronounced changes were found for lipids, choline containing compounds, glutamate and glutathione, nucleotide sugars, lactate, and some amino acids. Possible contributions of these metabolites to physiologic processes are discussed. The time-dependent metabolic response patterns suggest that A2780 cells on one hand and HEK-293 cells and A2780cisR cells on the other hand may follow different cell death pathways and exist in different temporal stages thereof.

## Introduction

Following the success of platinum-based anticancer drugs, with cisplatin [1] being the most widely used compound in this field [2], much attention has been given to ruthenium complexes as alternative agents to overcome some of the drawbacks associated with platinum-based treatment such as general toxicity, drug resistance or low selectivity [3,4]. Different types of ruthenium based complexes have been developed as promising anticancer drug candidates. Two Ru(III) complexes, KP1019 (NKP1339) [5] and NAMI-A [6,7], both bearing imidazole and chloride ligands, have reached phase II clinical trials [8]. KP1019 is more effective against primary tumors, while NAMI-A is more effective against metastasis, and both exhibit an increased selectivity thus leading to fewer side effects [9]. Half-sandwich Ru(II) complexes have also emerged as potent drug candidates [3]. For instance, the RAPTA complex family [10] has proven to be very promising and one of these complexes, RAPTA-C, has successfully completed preclinical trials [11].

**Fig 1:**
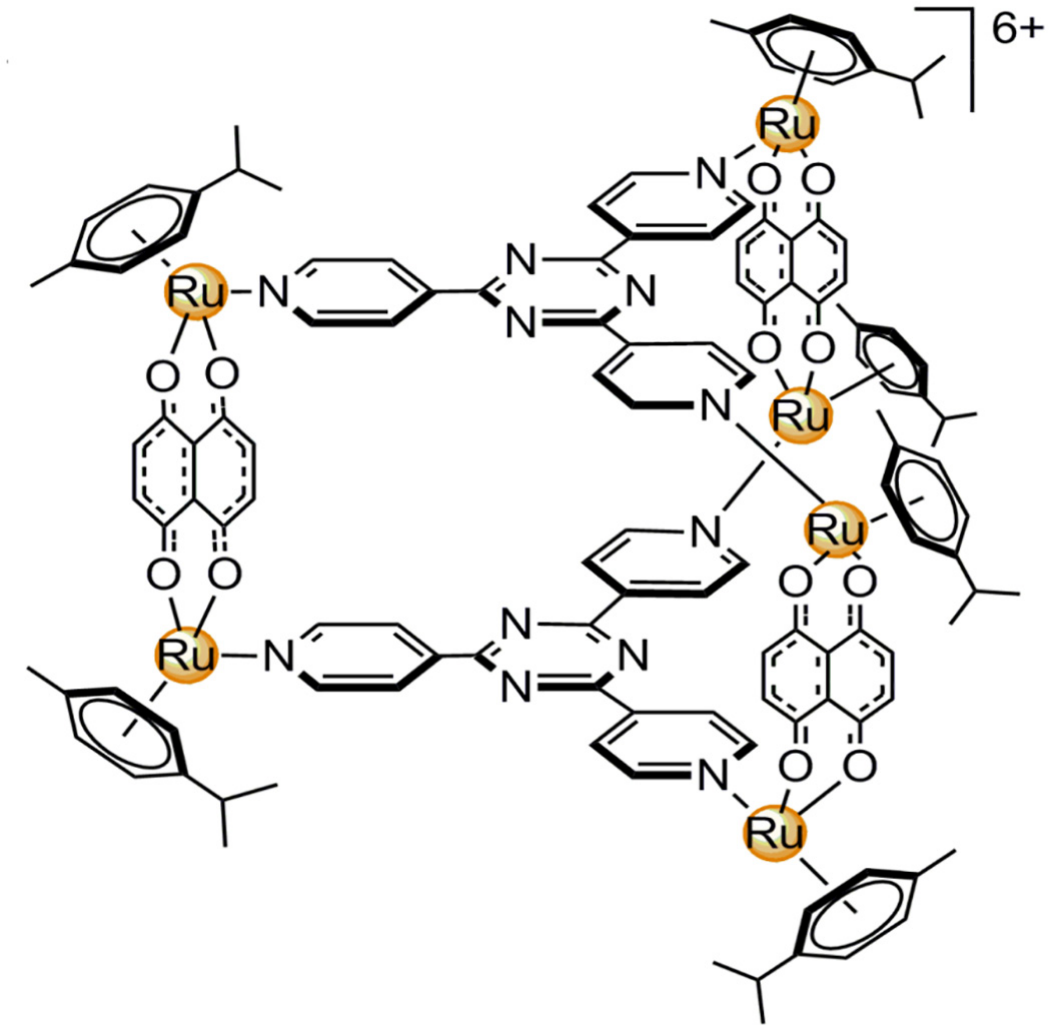
Ruthenium metallaprism [1]^6+^. Structure of the metallaprism [**1**]^6+^ used for cell incubation [(*p*-cymene)_6_Ru_6_(tpt)_2_(dhnq)_3_]^6+^, isolated as its triflate salt.

In our group, a series of water-soluble hexacationic arene ruthenium prisms have been prepared and probed for their cytotoxic activity and interactions with biological ligands [12–15]. This class of complexes exhibits several favorable properties as potential anticancer drugs: (i) their multiple positive charge improves water solubility and most likely also cell uptake, (ii) they exhibit remarkable low IC_50_ values [16], (iii) as large supramolecular complexes the enhanced permeability and retention (EPR) associated with most tumoral vascular systems [17] can lead to selective uptake, (iv) the cavity formed by the multinuclear ruthenium cages is capable to encapsulate guest molecules such as Pt- or Pd-acetylacetonate complexes [18,19] making drug delivery possible as well as synergistic effects by combining two active compounds. In this study, we report on the hexacationic ruthenium metallaprism [*p*-cymene)_6_Ru_6_(tpt)_2_(dhnq)_3_](CF_3_SO_3_)_6_, [**1**]^6+^, shown in Fig 1, and its effect on the metabolic profile of cancerous and non-cancerous cultured cells.

Although ruthenium complexes share some structural elements with platinum drugs, the mechanisms of action for most of the ruthenium complexes seem to follow different pathways which are not yet fully understood [20]. For instance, the half-sandwich complex RAPTA-C interacts and forms adducts with proteins like histones [21–23] while NKP1339 is most likely binding to DNA [20] and multiple interactions with various biomolecules have been identified for NAMI-A. The supposed targets and modes of actions have been recently reviewed for the most important compounds representing the various ruthenium complex classes with anti-cancer potential [20]. Considering the complexity of intracellular pathways, multiple targets may be involved in the anti-proliferative activity of these compounds. Monitoring simultaneously a broad range of cell metabolites, which are related to these pathways, is expected to provide useful information on the molecular mechanisms of action.

Therefore, in this study, a metabolomic approach for characterizing the effect of the ruthenium metallaprism [**1**]^6+^ (Fig 1) was used. The metabolic response of living cells following treatment with the ruthenium drug was directly analysed applying ^1^H high resolution magic angle spinning (HR-MAS) NMR spectroscopy. In recent years, HR-MAS NMR has emerged as a powerful non-invasive tool for recording the profile of small metabolites in biological material such as cells or tissues [24,25]. It has been successfully applied to identify and study biomarkers for disease and drug treatment [26,27]. In particular, several HR-MAS NMR based metabolomic studies on cells have proved to provide useful information on the cellular response to drug exposures like cisplatin [28–31], doxorubicin and methotrexate [32], tamoxifen [33], or docetaxel [34] under various conditions such as different incubation times or drug concentrations.

## Materials and Methods

### Synthesis of the ruthenium metallaprism

The hexanuclear ruthenium metallaprism [(*p*-cymene)_6_Ru_6_(tpt)_2_(dhnq)_3_](CF_3_SO_3_)_6_ (= [**1**](CF_3_SO_3_)_6_) with tpt = 2,4,6-tri(pyridin-4-yl)-1,3,5-triazine and dhnq = 5,8-dihydroxy-1,4-naphthoquinonato was prepared as previously described [19]. The structure is shown in Fig 1.

### Cell cultures

#### Cell material and cytotoxicity test

Human ovarian carcinoma cells A2780 and A2780cisR were obtained from the European Centre of Cell Cultures (ECACC, Salisbury, UK, catalogue No. 93112519 and 93112517, respectively); the human embryonic kidney cells HEK-293 were kindly provided by the group of Prof. Mühlemann, University of Bern. All cells were maintained in culture as described by the provider. The human ovarian cells were routinely grown in RPMI-1640 medium, which contained 10% fetal calf serum (FCS), 2 mM glutamine (Gln) and 1% antibiotics (penicillin/streptomycin), at 37°C and 5% CO_2_. HEK-293 cells were routinely grown in Dulbecco’s Modified Eagle Medium (DMEM), containing the same supplements as the RPMI-1640 medium and additionally 1% HEPES pH buffer. Cytotoxicity was determined using the Cell Counting Kit-8 (Dojindo Inc., EU GmbH). For this, the cells were seeded in 96-well plates as monolayers with 100 μL of cell solution (approximately 10,000 cells) per well. The Ru-complex was dissolved in DMSO serving as a vehicle to solubilize the complex, then taken up in the culture medium and further serially diluted to the appropriate concentration, to give a final DMSO concentration of 1%. 100 μL of drug solution was added to each well and the plates were incubated for 96 h. After incubation the culture medium was removed completely and subsequently, 10 μL kit solution and 100 μL fresh medium were added to the cells. The plates were incubated for another 90 min. The optical density, directly proportional to the number of surviving cells, was quantified at 450 nm using a multiwell plate reader and the fraction of surviving cells was calculated relative to the absorbance of untreated control cells. Evaluation was based on means from four independent experiments, each comprising four microcultures per concentration level.

#### Cell growth and exposure to the Ru-complex

All cells were seeded in 25 cm^2^ culture flasks with a density of approximately 8 x 10^4^ cells/cm^2^ and kept at 37°C in a humidified incubator in the presence of 5% CO_2_ for 24 h. After cell adhering, the experiment was initiated. The cell culture medium was removed completely. Subsequently, 5 mL fresh culture medium were added as well as the appropriate amount of a stock solution of [**1**](CF_3_SO_3_)_6_ in DMSO (c = 1 mM) to reach a final drug concentration of 0.9 μM for A2780 cells, 0.84 μM for A2780cisR cells, and 0.44 μM for HEK-293 cells corresponding to c = 2*IC_50_ for each cell line. Cells cultured in medium containing the same amount of DMSO only were used as controls. As IC_50_ values were collected at 96 h drug incubation, exposure times to [**1**]^6+^ were chosen well below this value (24 h) and at a longer period of 72 h. At these two time points (t = 24 h and 72 h) the cells were harvested by trypsination and centrifuged at 2000 rcf for 5 min. The supernatant was discarded and the cells suspended in 1 mL freezing medium (50% FCS, 40% culture medium and 10% DMSO). The freezing medium acts as cryoprotectant allowing for maintenance of cell integrity [35]. After transfer into cryo vials, the samples were frozen and stored at -80°C. For each cell line and each time point (24 h and 72 h), 8 control samples and 10 drug incubated samples were prepared yielding a total of 108 cell samples, each containing approximately 1–5*10^6^ cells for NMR analysis.

#### Trypan blue exclusion assay

At each time point, the amount of living cells was assessed with the Trypan blue exclusion assay to yield the number of living versus dead cells. This assay can be used as a measure of cell membrane integrity. For the assay, the cells were briefly incubated with a solution of 0.4% (w/v) Trypan blue dye in PBS and counted in an automated cell counter (Countess automated cell counter, Life Technologies Europe B.V.). Cell viability was expressed as the percentage of living cells (dye-excluding) relative to the total number of cells. The cell viabilities of the thawed cell samples right before NMR measurement were found to be between 84% and 99% for all three cell lines and were not significantly different from freshly harvested cells.

#### Sample preparation for NMR measurements

Before NMR analysis, each frozen cell sample was thawed at 37°C, transferred into 2.5 mL preheated culture medium and centrifuged at 2000 rcf for 5 min. The supernatant was discarded and the cells were washed three times with 1 mL PBS. After the last washing step, the cells were taken up in 20 μL 10 mM D_2_O-based PBS (pH 7.4). The cell suspension was transferred into a standard 4 mm MAS rotor using a 12 μL insert. All cell samples were measured in a random order. For each cell line, the NMR spectral profile was unaffected by the freezing and thawing procedure as compared to freshly harvested cells (see S1 Fig).

### ^1^H High Resolution Magic Angle Spinning (HR-MAS) NMR spectroscopy

#### Acquisition

The ^1^H HR-MAS NMR experiments were performed on a Bruker Avance II spectrometer (Bruker BioSpin) operating at a resonance frequency of 500.13 MHz for ^1^H. The instrument is equipped with a 4 mm HR-MAS dual inverse ^1^H / ^13^C probe (Bruker BioSpin) with a Magic Angle gradient. All experiments were carried out at a magic angle (54.7°) spinning rate of 3 kHz and a stabilized temperature of 310 K. The Bruker Topspin software (version 2.1, patch level 5) was used to acquire the NMR data. For each sample, two 1D ^1^H HR-MAS NMR spectra were recorded both with presaturation of the water resonance applying a 1D NOESY and a 1D cpmg sequence for suppressing resonances with short *T*_2_ relaxation times (“*noesypr1d*” and “*cpmgpr1d*” respectively from the Bruker pulse-program library). Each 1D ^1^H NMR spectrum was acquired applying 512 (for noesy1d) or 1024 (for cpmg1d) transients, a spectral width of 6002.4 Hz, a data size of 32 K points, an acquisition time of 2.73 s, and a relaxation delay of 4 s (for noesy1d) or 2s (for cpmg1d). The 90° pulse length was 8.7 *μ*s, the noesy mixing time 10 ms and the echo time applied for the cpmg-spectra was 9.6 ms (16 loops, rotor-synchronized interpulse delay of 0.3 ms). The total experiment time for acquiring both 1D spectra was about 2.5 h. During this time period, MAS spinning at 3kHz had little effect on the NMR spectroscopic profile of the cell samples as could be established from preceding time-dependent measurements (S2 Fig). In addition, all samples were measured following a strict timely constant protocol. For selected samples (from both, the control and drug-treated groups at each time point) phase-sensitive 2D ^1^H^1^H-TOCSY spectra using the DIPSI2 sequence for mixing and 2D J-resolved spectra (*“dipsi2phpr”* and *“jresgpprqf”*, respectively, from the Bruker pulse program library) both with presaturation during relaxation delay were recorded to help spectral assignment. Representative 2D ^1^H^1^H-TOCSY and ^1^H-*J*-resolved spectra are shown in S3–S6 Figs.

#### Spectral processing

Spectral processing was performed using the Bruker Topspin software (version 3.2, patch level 3). The co-added free induction decays (FIDs) were exponentially weighted with a line broadening factor of 1.0 Hz, Fourier-transformed, and manually phase corrected to obtain the ^1^H NMR spectra. Each spectrum was manually baseline corrected using a cubic spline interpolation method according to selected baseline points. Chemical shifts were referenced to the phosphocholine (PC) signal at δ = 3.23 ppm for A2780 and HEK-293 cell samples and to the creatine (Cre) signal at δ = 3.05 ppm for the A2780cisR cell samples.

### Data analysis

Multi- and univariate statistical analyses were performed using MATLAB R2012a (Mathworks), PLS-Toolbox 7.5.2 (Eigenvector Research, Inc.), and Excel 2010 (Microsoft). Prior to data analysis, four ^1^H HR-MAS NMR cell spectra were excluded out of the total of 108 spectra, because their signal to noise ratio was too low leaving a total of 104 spectra. The 1D NOESY spectra were used for multivariate analysis of the spectral regions between 0 and 6.5 ppm, while single resonances in the spectral region between 5 and 9 ppm were evaluated based on the 1D cpmg spectra.

#### Multivariate analysis of 1D NOESY spectra

The 104 spectra were subdivided into 97 individually sized buckets within the range between 0.5 and 6.2 ppm. The buckets were selected on an overlay of all averaged spectra according to single resonances or regions of overlapping resonances. The average bucket width was 0.035 ppm (min 0.011 ppm, max 0.139 ppm). Noise regions, the region of the residual water resonance (4.3–4.9 ppm), and the regions of ethanol (1.15–1.25 ppm) and DMSO (2.69–2.78 ppm) deriving from contaminations of cell work-up were excluded. Bucket selection is illustrated in S7 Fig. To account for differences in sample weight probabilistic quotient normalization (PQN) [36] was applied to the bucket integrals. The variables (buckets) were mean centered and scaled to unit variance, which results in equal weight of high and low intensity signals. For multivariate statistical analyses all spectra were grouped according to cell line (A2780, A2780cisR, and HEK-293), incubation time (24 h and 72 h) and treatment (drug incubation and control) resulting in 12 subgroups. In a first step, principle component analysis (PCA) was applied to the data to test for clustering (S8 Fig). Partial least squares discriminant analysis (PLS-DA) was then applied to each pair of control and drug-treated cell data sets for probing separation of the corresponding classes. The results were cross-validated using the “leave-one-out” method. For interpretation of the PLS-DA plots, the load values of the first PLS-component for each PLS-plot were determined and plotted as function of bucket. Buckets with load values beyond an arbitrary threshold of +0.1 and—0.1 were considered strong contributors to group discriminations. The normalized integrals of these corresponding buckets were further analysed. To compare the effect of drug incubation as function of incubation time and cell line, relative differences for 15 averaged and normalized bucket integrals I_drug_—I_ctrl_ were calculated and plotted. To probe for significance of differences, two-tailed unpaired two sample student’s t-test was applied to the corresponding bucket integrals. All p-values were multiplied with a factor of 15 to correct for multiple comparisons (Bonferroni correction). A p-value < 0.05 (confidence level 95%) was considered statistically significant.

#### Univariate analysis of 1D cpmg spectra

In the spectral region between 5 and 9 ppm of the 1D cpmg spectra integrals of resonances deriving from 11 different compounds were evaluated. The integrals were normalized by probabilistic quotient normalization (PQN) and for each peak integral the mean value and standard deviation were calculated. The difference between the means of control and drug treated groups for each cell line and each incubation time (24 h, 72 h) was assessed using the t-test. A p-value < 0.05 (confidence level 95%) was considered statistically significant. All p-values were multiplied with a factor of 11 to correct for multiple comparisons (Bonferroni correction).

## Results and Discussion

### ^1^H HR-MAS NMR spectra of cells

**Fig 2:**
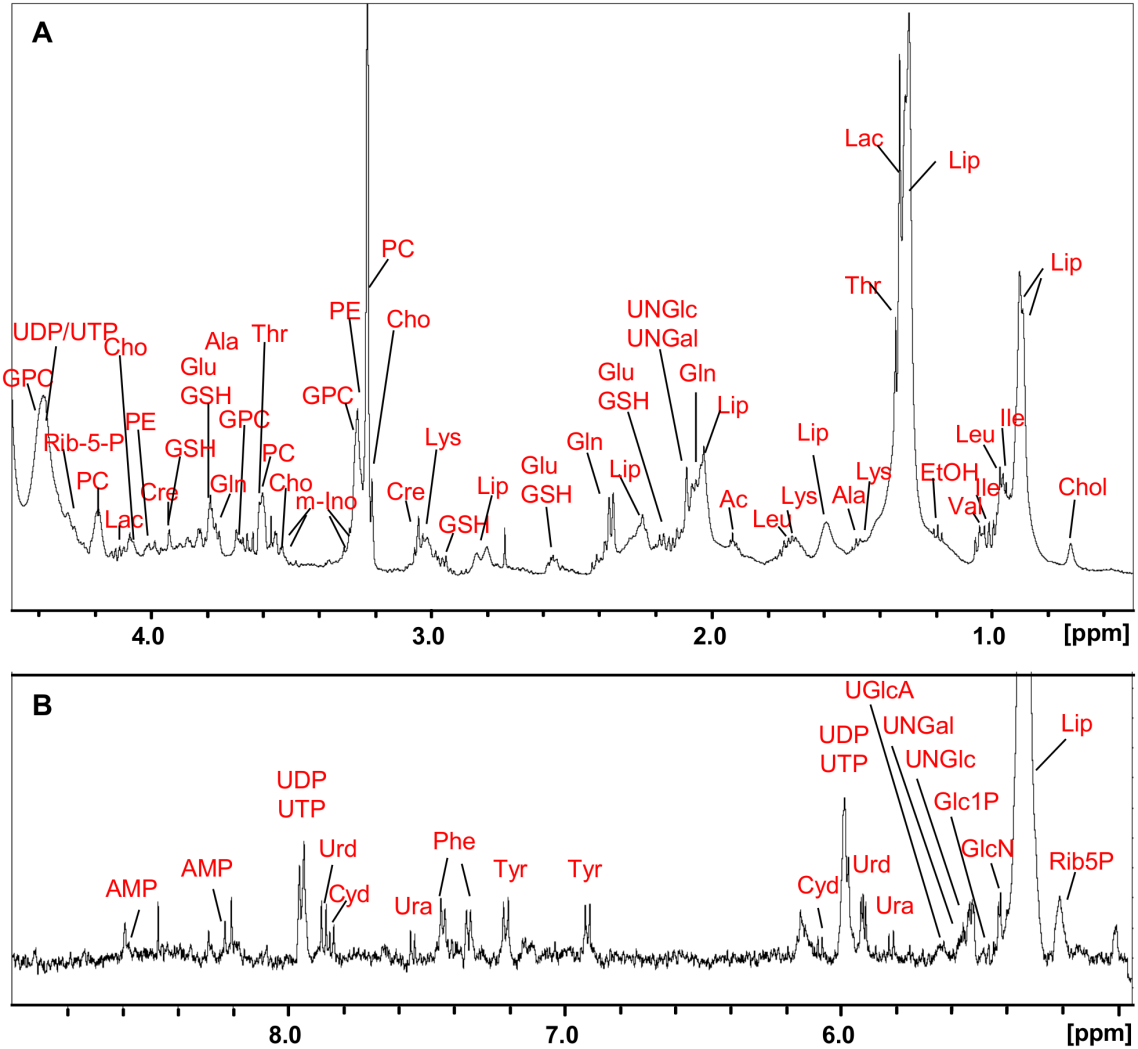
^1^H HR-MAS NMR spectrum of cells. ^1^H HR-MAS-cpmg spectrum of a cell suspension (A2780) in PBS with resonance assignments according to Table 1. **A**: aliphatic region (0.5–4.5 ppm), **B**: aromatic region (5–9 ppm, scaled up ~*4).

In Fig 2, a representative ^1^H HR-MAS NMR spectrum with some of the resonances assigned to specific cell metabolites is shown for a suspension of A2780 human ovarian carcinoma cells in phosphate buffered saline (PBS). A complete list of all the resonances, which were assigned to cellular metabolites, is summarized in Table 1. The assignment was based on 2D ^1^H^1^H TOCSY and *J*-resolved spectra (S3–S6 Figs) with the TOCSY cross peaks given in Table 1, as well as on spectral reference data and spiking experiments, data derived from the literature [35,37,38] and the human metabolome data base (HMDB) [39] with a match of about ± 0.02 ppm. While resonance positions can depend on factors such as pH, solvent and ionic strength [40,41], the majority of NMR data derived from biological samples is collected in physiological solutions, i.e. with isotonic buffer at neutral pH. Therefore, there is typically good agreement in chemical shift values reported for small metabolites from biological material in the literature or databases [42,43]. Furthermore, it could be shown that metabolite differences comparing whole cells and cell extracts mainly appeared in ratios rather than in peak positions of the corresponding NMR spectra [35]. In the spectral region between 0 and 4.5 ppm (Fig 2) a major contribution of the signals can be ascribed to lipid components such as the lipid ω-CH_3_ resonance around 0.9 ppm and the lipid—(CH_2_)_n_ methylene resonance around 1.3 ppm. The spectrum was acquired with a *cpmg* sequence (*cpmgpr1d*) applying a *T*_2_-relaxation filter (TE = 9.6 ms) to suppress broad components originating from large molecules. This filtering procedure was necessary for the analysis of the aromatic spectral region with low signal contributions, while for the aliphatic spectral region the unfiltered spectra (*noesypr1d*) were used. The NMR visibility of the lipid signals even with a weak *T*_2_-filter indicates that they derive from mobile lipids. These mobile lipids mainly originate from intracellular lipid droplets rather than from membranes where only small lipid microdomains may account for a smaller fraction of NMR visible lipids [44]. Further strong contributions also associated with the lipid metabolism can be ascribed to the resonances from choline containing compounds with their—N^+^(CH_3_)_3_ groups appearing around 3.2 ppm. Additionally, resonances of small metabolites typically occurring in cell spectra [35,37] like amino acids, glutathione (GSH), and creatine (Cre) are assigned in the spectral region between 0 and 4.5 ppm. In particular, most of the assigned signals in the highly overlapping region between 3 and 4.5 ppm—like for example the α-CH protons of the amino acids—were based on the analysis of additional 2D TOCSY cross peaks.

**Table 1:** Signal assignment of protons from A2780 ov. cancer cell suspension (PBS).

Peak	Chemical shift [ppm]	Compound	TOCSY [ppm]	Group
1	0.723	Cholesterol & esters (Chol)		-CH_3_
2	0.893	Lipid (Lip)	1.55	ω-CH_3_
3	0.904	Lipid (Lip)	1.308	ω-CH_3_
4	0.96 (t)	Isoleucine (Ile)	1.275,1.476	δ-CH_3_
5	0.97 (d)	Leucine (Leu)	1.73	δ-CH_3_
6	1.021 (d)	Isoleucine (Ile)	2.0	β’-CH_3_
7	1.037	Valine (Val)	2.274	γ-CH_3_
8	1.195 (t)	Ethanol (EtOH)	3.66	-CH_3_
9	1.30 (br)	Lipid (Lip)	0.904	(-CH_2_)_n_
10	1.318 (br)	Lipid (Lip)	1.595, 2.031, 2.245, 5.33	(-CH_2_)_n_
11	1.324 (d)	Lactate (Lac)	4.118	β-CH_3_
12	1.34 (d)	Threonine (Thr)	4.273	γ-CH_3_
13	1.47	Lysine (Lys)	1.73, 3.02	γ-CH_2_
14	1.48	Alanine (Ala)	3.79	β-CH_3_
15	1.595 (br)	Lipid (Lip)	1.318, 2.245	β-CH_2_
16	1.72	Lysine (Lys)	1.47, 3.02	β-,δ- CH_2_
17	1.73	Leucine (Leu)	0.97	γ-CH_2_
18	1.928 (s)	Acetate (Ac)		-CH_3_
19	2.035 (br)	Lipid (Lip)	1.32, 5.33	-CH_2_-CH =
20	2.06	Glutamine (Gln)	2.36	β-CH_2_
21	2.092 (s)	UDP-N-Acetyl-glucosamine /galactosamine (UNGlc, UNGal)		-CH_3_
22	2.18 (m)	Glutathione (GSH)	2.57, 3.8	β-CH_2_(Glu)
23	2.18 (m)	Glutamate (Glu)	2.57, 3.8	β-CH_2_
24	2.245 (br)	Lipid (Lip)	1.32, 1.6	α-CH_2_
25	2.36 (m)	Glutamine (Gln)	2.06, 3.78	γ-CH_2_
26	2.57	Glutathione (GSH)	2.18, 3.8	γ-CH_2_(Glu)
27	2.57	Glutamate (Glu)	2.18, 3.8	γ-CH_2_
28	2.815	Lipid (Lip)	5.33	= CH-CH_2_-CH =
29	2.96	Glutathione (GSH)	4.585	β-CH_2_(Cys)
30	3.02	Lysine (Lys)	1.47, 1.72	ε-CH_2_
31	3.05	Creatine (Cre)		-CH_3_
32	3.21	Choline (Cho)		-N^+^(CH_3_)_3_
33	3.23	Phosphocholine (PC)		-N^+^(CH_3_)_3_
34	3.24	Phosphoethanolamine (PE)	4.0	-OCH_2_
35	3.27	Glycerophosphocholine (GPC)		-N^+^(CH_3_)_3_
36	3.28	myo-Inositol (m-Ino)	3.55	-CH
37	3.53	Choline (Cho)	4.07	-CH_2_
38	3.55	myo-Inositol (m-Ino)	3.28	-CH
39	3.6	Phosphocholine (PC)	4.2	-CH_2_
40	3.605	Threonine (Thr)	1.345	-CH
41	3.68	Glycerophosphocholine (GPC)	4.38	-CH_2_
42	3.78	Glutamine (Gln)	2.36, 2.06	α-CH
43	3.784	Alanine (Ala)	1.48	α-CH
44	3.8	Glutathione (GSH)	2.57, 2.18	α-CH(Glu)
45	3.8	Glutamate (Glu)	2.57, 2.18	α-CH
46	3.938	Glutathione (GSH)		-CH_2_(Gly)
47	3.94	Creatine (Cre)		-CH_2_
48	3.96	Tyrosine (Tyr)	3.22, 3.09	α-CH
49	4.0	Phosphoethanolamine (PE)	3.24	-CH_2_
50	4.07	Choline (Cho)	3.53	-CH_2_
51	4.118 (q)	Lactate (Lac)	1.324	α-CH
52	4.2	Phosphocholine (PC)	3.6	-CH_2_
53	4.28	Ribose-5-phosphate (Rib5P)	5.21	-CH
54	4.38	UDP/UTP	5.99	-CH (Rib)
55	4.38	Glycerophosphocholine (GPC)	3.68	-CH_2_
56	5.21	Ribose-5-phosphate (Rib5P)	4.28, 4.09	-CH
57	5.33	Lipid (Lip)	2.82, 2.04, 1.32	-CH = CH
58	5.43 (d)	Glucosamine (GlcN)	3.59	-CH-1
59	5.46 (dd)	α-Glucose-1-phosphate (Glc1P)		-CH-1 (Glc)
60	5.55 (dd)	UDP-N-Acetyl-glucosamine (UNGlc)		-CH-1 (Glc)
61	5.59 (dd)	UDP-N-Acetyl-galactosamine (UNGal)		-CH-1 (Gal)
62	5.67 (dd)	UDP-Glucuronic acid (UGlcA)		-CH-1 (GlcA)
63	5.82 (d)	Uracil (Ura)	7.55	-CH
64	5.92 (2xd)	Uridine (Urd)	4.35, 7.86	-CH
65	5.98 (d)	UDP/UTP	7.95	-CH (Ura)
66	5.99 (d)	UDP/UTP	4.38	-CH (Rib)
67	6.07 (d)	Cytidine (Cyd)	7.84	-CH
68	6.92 (d)	Tyrosine (Tyr)	7.21	-CH
69	7.21 (d)	Tyrosine (Tyr)	6.92	-CH
70	7.35 (d)	Phenylalanine (Phe)	7.44	-CH
71	7.44 (d)	Phenylalanine (Phe)	7.35	-CH
72	7.55 (d)	Uracil (Ura)	5.82	-CH
73	7.84 (d)	Cytidine (Cyd)	6.07	-CH
74	7.86 (d)	Uridine (Urd)	5.9	-CH
75	7.95 (d)	UDP/UTP	5.99	-CH (Ura)
76	8.23	AMP		-CH
77	8.58	AMP		-CH

Resonances with much lower intensity except for the relatively strong—CH = CH- signal of unsaturated lipids were detected in the spectral region between 5 and 9 ppm (Fig 2B). They mainly derive from aromatic amino acids like tyrosine (Tyr) and phenylalanine (Phe), nucleotides or the anomeric protons of sugar components, especially nucleotide sugars [28,45]. The corresponding acetyl-methyl group of the N-acetylated nucleotide sugars gives rise to a clearly visible singlet at 2.09 ppm in the aliphatic region (Fig 2A), as was confirmed by spiking experiments.

### The metabolic profile of untreated cells—PCA of control cells

The aim of the present study was to probe the effect of the ruthenium metallaprism [**1**]^6+^ (Fig 1) onto 3 different cell lines covering both cancer cells (A2780) and non-cancer cells (HEK-293), as well as cancer cells which have developed a resistance towards cisplatin treatment (A2780cisR). For this, cells treated with [**1**]^6+^ were compared to untreated control cells.

**Fig 3:**
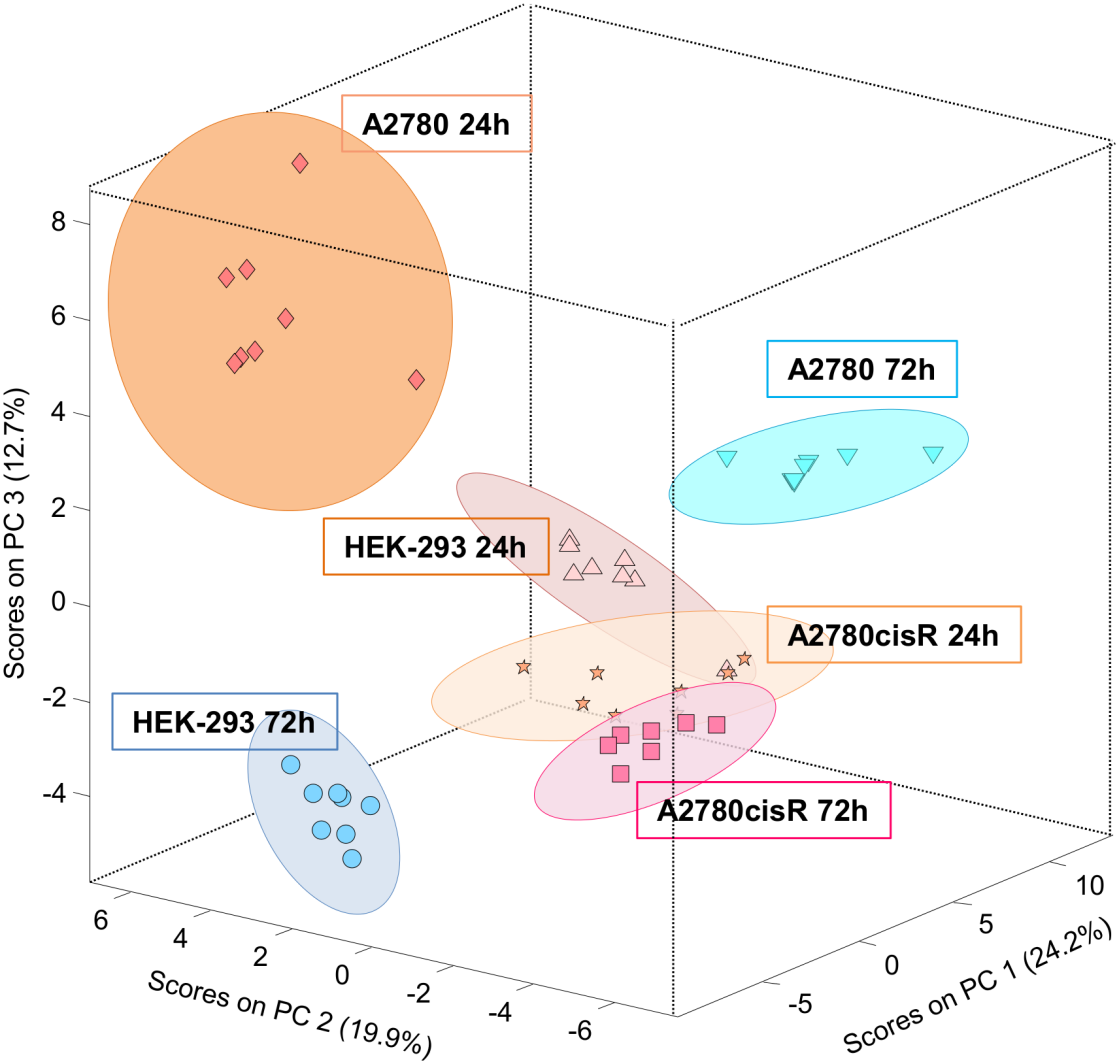
PCA scores plot for all control samples. PCA scores plot (PC 1—PC 3) for all cell samples A2780, A2780cisR and HEK-293 at incubation times of 24h and 72h.

Principal component analysis (PCA) was applied to the ^1^H HR-MAS spectra of all control samples derived from the three different cell lines and 2 different growth times, 24 h and 72 h, for each cell line (on average, 8 samples per group). Spectral regions between 0 and 6.4 ppm from the 1D *noesy* spectra were taken as basis for the analysis. The corresponding PCA scores plot for the first 3 principal components, explaining 56.8% of the variance, is shown in Fig 3. A clear clustering was observed not just for each individual cell line but also for the different growth durations within each cell line. Since PCA is an unsupervised method the clustering demonstrates a good reproducibility of the corresponding HR-MAS cell spectra. Each cell line is characterized by its specific metabolite spectrum due to different metabolite ratios. Accordingly, this also demonstrates that proton HR-MAS NMR spectra of cells can be used for chemometric phenotyping based on their specific metabolic fingerprint as has been previously shown in the literature [34,37,46,47]. The differentiation between the two cancer cell lines A2780 and A2780cisR on one hand and the normal HEK-293 cell line on the other hand is not surprising, since metabolic alterations driven by oncogenic signaling are responsible for cell growth and proliferation in cancer [48]. One of the features in cancer cells among others is an increased lipid biosynthesis, and accordingly an overall increase in lipid signals was also the main contributor for discriminating A2780 cells from HEK-293 cells in partial least squares discriminant analysis (PLS-DA, S9 and S10 Figs). The cisplatin resistance of A2780cisR cells has been reported to be correlated with increased levels of glutathione as compared to cisplatin sensitive cells [49]. Here, A2780cisR cells were rather mainly distinguished by increased lactate, several amino acids and uridine levels (S9 and S10 Figs).

**Fig 4:**
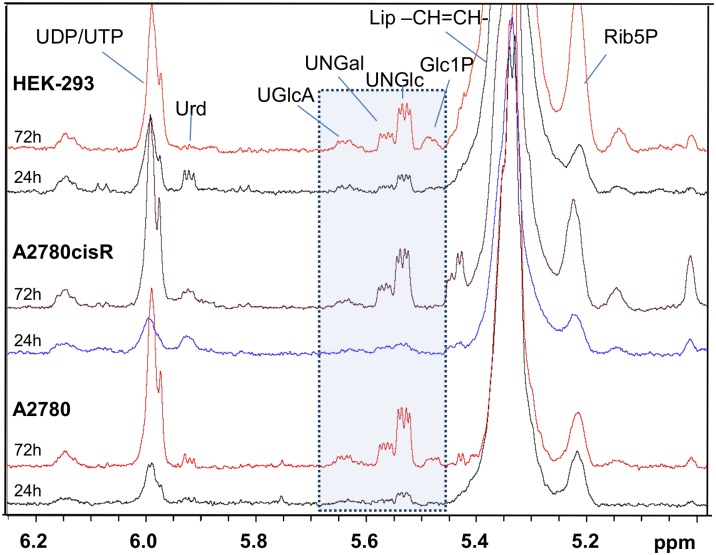
^1^H HR-MAS NMR spectral region of phosphate sugars. Spectral region 5–6.3 ppm for averaged ^1^H HR-MAS spectra of control cell samples A2780, A2780cisR and HEK-293 at incubation times of 24h and 72h. Region with resonances of phosphate sugars Glc1P, UNGlc, UNGal, and UGlcA is highlighted.

Interestingly, a clear distinction could not just be observed for the different cell types but also for cells of the same cell line obtained from two different growth periods of 24 h and 72 h (Fig 3 and S11–S13 Figs). This suggests that the cells undergo a metabolic drift over time. While after 72 h the cells are in the logarithmic growth phase, their cell density in culture has increased at this time point, but not reached confluence according to visual inspection. Confluence is reported to be reached on average after 80 h, 120 h and 140 h for HEK-293, A2780 and A2780cisR cells, respectively [50]. Therefore, both, enhanced cell density and nutrient consumption from the growth medium may be reflected in the altered metabolic profiles. Among the components which were clearly increased after 72 h in all three cell lines are the nucleotides UDP and UTP and the corresponding N-acetylated glucose (UNGlc) and galactose (UNGal) sugars as is depicted in Fig 4. Increase of these phosphate compounds indicates that the cellular energy metabolism is affected at longer growth periods most likely reflecting adaptive processes. In agreement with our finding, increasing levels of N-acetylated UDP sugars with progressive cell growth could be found in cultured MDCK cells as intermediates of glycogenesis for energy storage [51]. Cell density or different cell growth phases have also been reported to affect cell metabolism in various ways such as amino acids utilization [47], concentrations of myo-inositol and glutamine [52], mobile lipids, or choline containing compounds [53]. An overall increase of lipid signals accompanied by a relative decrease of choline containing compounds found at long incubation times (S11–S13 Figs) seems to indicate cell density or stress related slow-down of cellular growth [53,54]. As a consequence it is important to perform comparative studies of cellular drug response only on exactly matched cell samples grown under same conditions at equal incubation periods to account for the specific metabolic ground level state of the cultured cells. In this sense, our data are interpreted such that metabolic alterations in response to drug treatment observed after 24 h or 72 h incubation have to be related to the specific metabolic background of the particular growth phase at each time point.

### Toxicity of the metallaprism [1]^6+^against cells

The cytotoxicity of [**1**]^6+^ was tested against the three cell lines and compared to the toxicity of cisplatin assessed in the same experimental set-up. The corresponding IC_50_ values are given in Table 2. Compared to cisplatin, the cytotoxicity of [**1**]^6+^ was clearly higher against A2780 cancer cells with an IC_50_ value below 1 *μ*M. Moreover, [**1**]^6+^ has an IC_50_ value in a similar range against the cisplatin resistant cell line (A2780cisR) indicating that the cytotoxic action seems to bypass the resistance mechanism, which is effective against cisplatin. However, the low IC_50_ value of [**1**]^6+^ against HEK-293 cells indicates that the complex despite its potency to overcome resistance does not exhibit selectivity. Our previous studies have suggested that compared to analog large ruthenium complexes the high cytotoxicity of the present compound may be related to its relatively high stability towards biological ligands [12–15].

**Table 2:** IC_50_ values of [1](CF_3_SO_3_)_6_ and cisplatin determined for the three cell lines.

	IC_50_ [μM]	
Cell line	[1](CF_3_SO_3_)_6_	cisPlatin
**A2780**	0.450 ± 0.067	2.942 ± 0.168
**A2780cisR**	0.422 ± 0.007	21.90 ± 0.800
**HEK-293**	0.223 ± 0.013	86.67 ± 23.11

### Cellular response to drug treatment—Multivariate analysis for the spectral region 0–6.4 ppm

To probe the effect of incubation during 24 h and 72 h with [**1**]^6+^ onto the metabolic profile of the three different cell lines, the spectral regions between 0 ppm and 6.4 ppm of the *noesy1d* HR-MAS spectra were divided into 97 individually sized buckets and evaluated by multivariate analysis. For this, the data were combined to 6 pairs of control and drug-treated sample sets according to the 3 cell lines and 2 incubation times used in this study. PCA applied to the 6 data sets resulted in a complete separation of control and drug-treated samples for each group (S8 Fig). For all groups except HEK-293 cells at 24 h, the separation could be achieved solely along the first principal component PC-1 (S8A–S8D and S8F Fig). On average, clustering appeared stronger for drug-treated samples as compared to controls indicating that the drug-treatment seems to impose spectral features making them more similar on top of their inherent variability, which gives rise to larger scattering in untreated cell samples.

**Fig 5:**
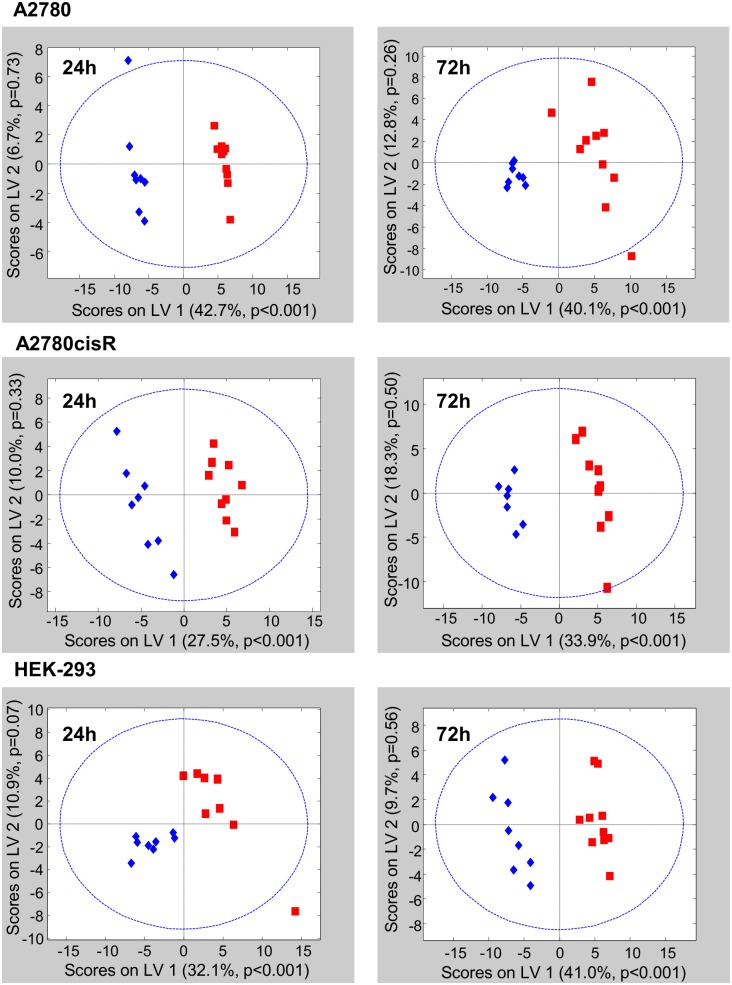
PLS-DA of control and drug-treated cells. PLS-DA scores plot (LV 1—LV 2) comparing control (blue) and drug treated (red) cell samples for the 3 different cell types A2780, A2780cisR and HEK-293 at incubation times of 24h and 72h. ∙∙∙∙∙∙∙∙∙ 95% Confidence level

To probe for discriminating features between control and drug treated cell samples, PLS-DA was applied to the 6 subgroups of cell spectra. The resulting PLS-scores plots are shown in Fig 5 for the first two PLS components (latent variables LV 1 and LV 2). Like in PCA, a clear distinction between control and drug-treated samples was obtained already along the first PLS component for each pair of samples derived from the 3 cell lines and 2 incubation times. Out of the total of 104 spectra, only 3 spectra were outside the 95% confidence level of the corresponding cross-validated PLS model (Fig 5).

**Fig 6:**
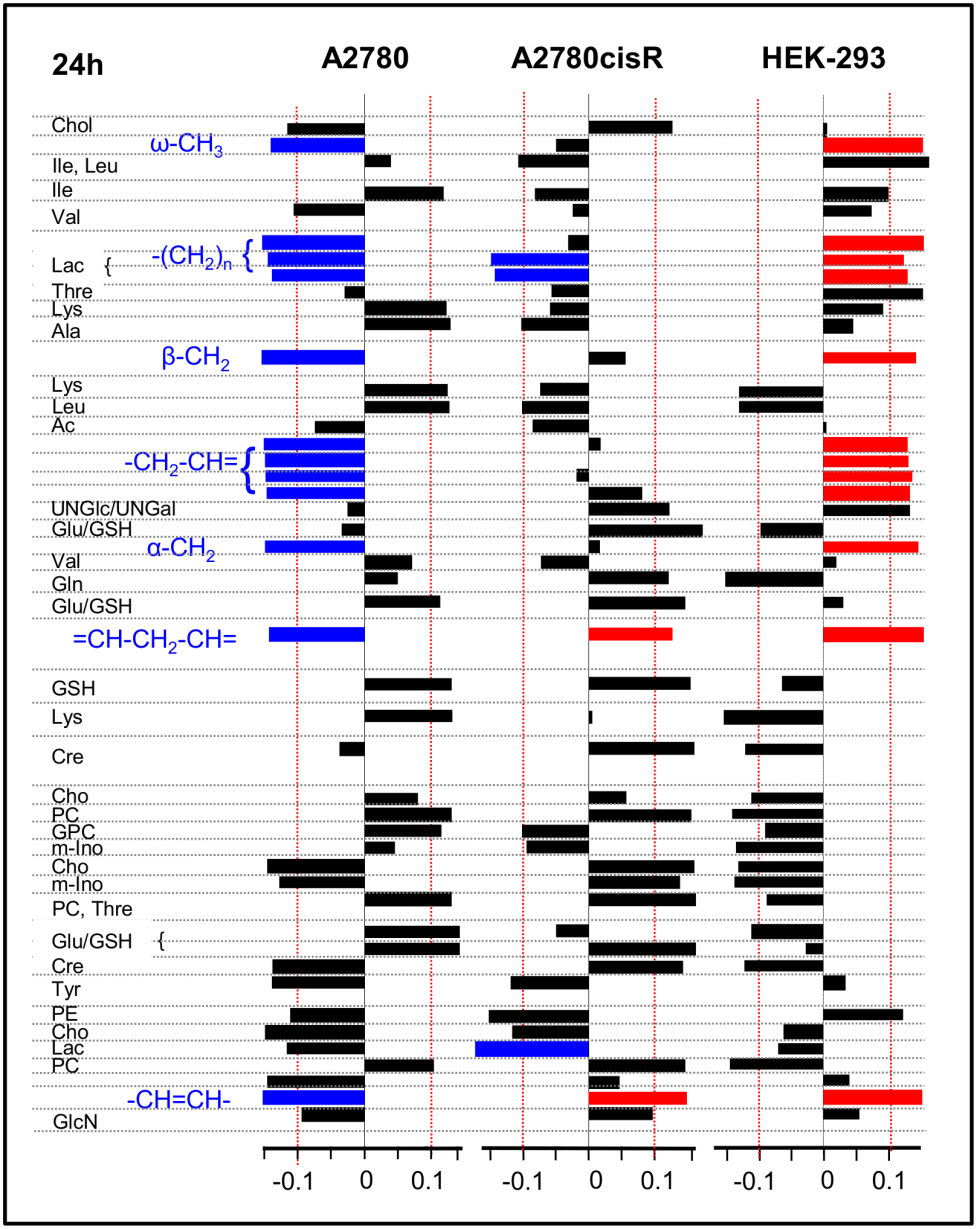
PLS-loadings (24 h). PLS-loadings of the first PLS component (LV 1) for the 3 cell lines A2780, A2780cisR and HEK-293 and 24h incubation time. Buckets are assigned to metabolites. Strong lipid contributions (and Lac for A2780cisR) are highlighted according to their sign (blue: < -0.1, red: > 0.1).

**Fig 7:**
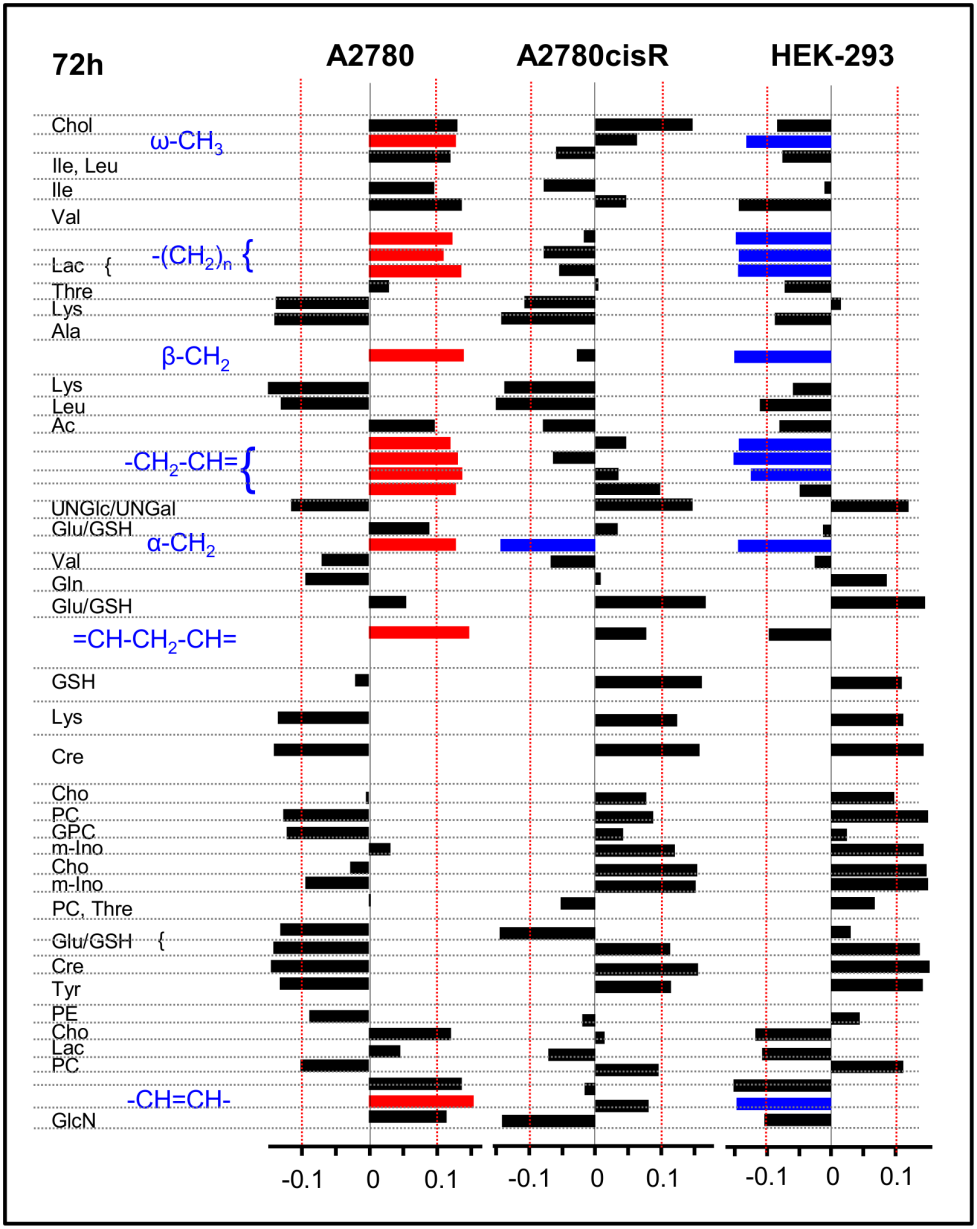
PLS-loadings (72 h). PLS-loadings of the first PLS component (LV 1) for the 3 cell lines A2780, A2780cisR and HEK-293 and 72h incubation time. Buckets are assigned to metabolites. Strong lipid contributions are highlighted according to their sign (blue: < -0.1, red: > 0.1).

The PLS load values were examined to identify which resonances or metabolites mainly contributed to the discrimination between the classes. The corresponding loading plots for the first latent variable (LV-1) are shown in Fig 6 for 24 h and in Fig 7 for 72 h incubation time. The bar plots display load values for buckets comprising resonances, which were assigned to a specific metabolite with abbreviations according to Table 1 (for complete loading plots, see S14—S16 Figs). Spectral regions with load values beyond an arbitrary threshold of + 0.1 and—0.1 indicated by a red line in Figs 6 and 7 were considered as regions with high influence on the separation between control and drug-treated samples. A specific metabolite was only assumed to be discriminating if the multiple resonance peaks of the corresponding metabolite exhibited consistent changes. These correlated changes help to interpret peak intensity changes deriving from overlapping regions. According to Fig 5, for all 6 discriminant analyses the control samples (shown in blue) had negative scores on LV-1, while the drug-treated samples (shown in red) had positive scores on LV-1. Thus, metabolites with resonances giving rise to negative load values were more expressed in control samples while metabolites with positive load values were more expressed in drug treated samples (Figs 6 and 7).

#### Changes in lipids and choline containing compounds

**Fig 8:**
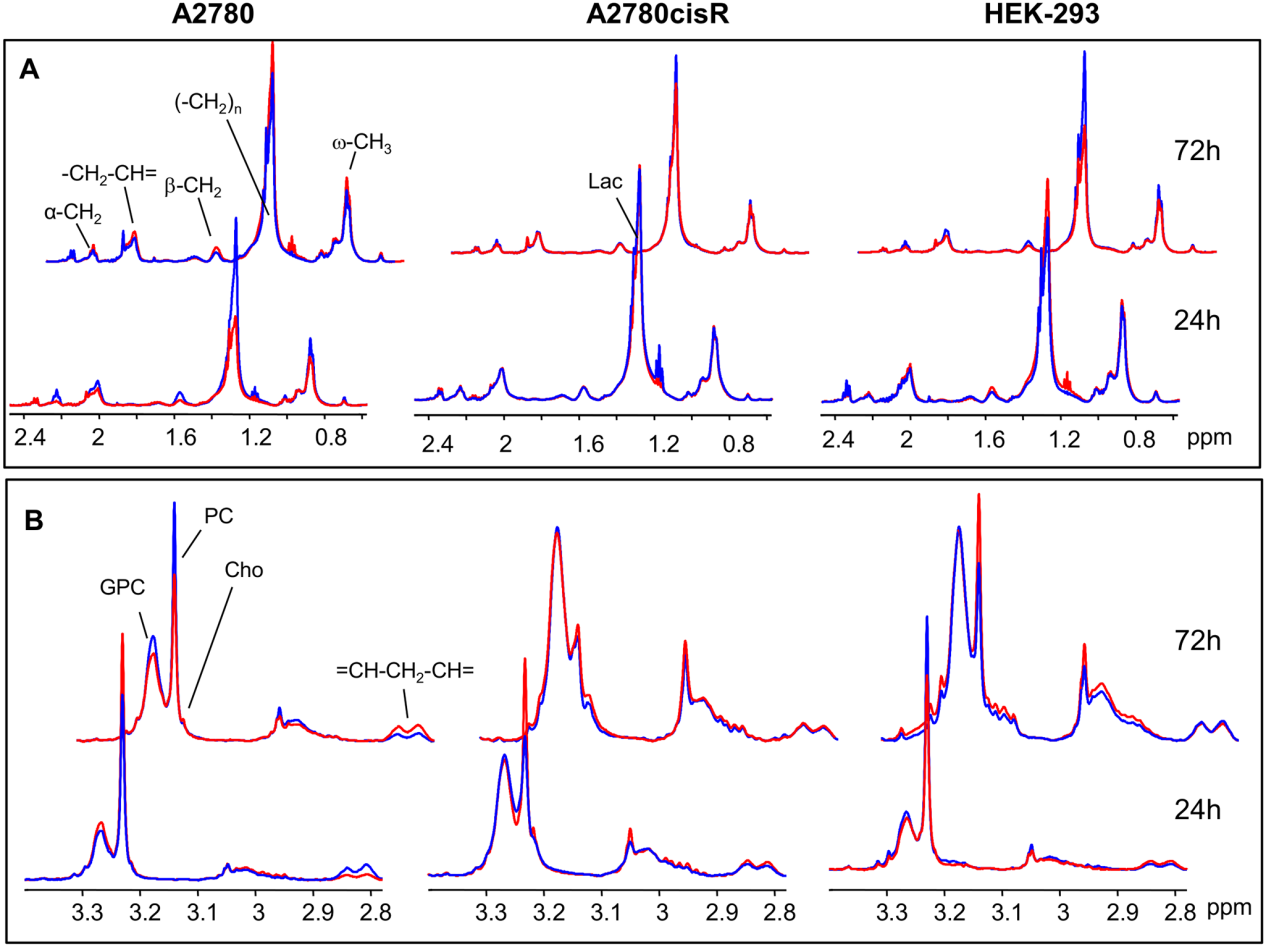
^1^H HR-MAS NMR mean spectra. PQN-normalized mean 1D noesy spectra for control (blue) and drug treated (red) cell samples for spectral regions of **(A)** saturated lipids and **(B)** choline containing compounds.

In Fig 8, normalized mean ^1^H HR-MAS spectra derived from control and drug-treated cell samples from both incubation times are shown for the spectral regions in which the main lipid signals (Fig 8A) and the choline containing compounds appear (Fig 8B). These spectra visualize the most pronounced changes found in PLS. In A2780 cancer cells, incubation with the Ru-complex mainly induced changes in lipids and choline containing compounds. After 24 h, there was a relative simultaneous decrease in all lipid resonances including the fatty acid ω-methyl, the methylene and the unsaturated fatty acid groups. At the same time, there was a concomitant increase in phosphocholine (PC) and glycerophosphocholine (GPC). After 72 h, the effect was inversed with increased lipid and decreased PC and GPC levels in response to drug treatment.

On the contrary, in cisplatin resistant A2780cisR cells a selective increase only in unsaturated lipids (-CH = CH- and = CH-CH_2_-CH = peaks) was observed after 24 h drug incubation, while the remaining lipid resonances did not contribute to separation from control cells (Fig 6). For the spectral region around 1.32 ppm, where lipid—(CH_2_)_n_ and lactate—CH_3_ signals overlap (Fig 8A), high bucket integrals in A2780cisR control samples were most likely due to increased lactate levels rather than lipid. This was supported by the covariant change observed for the corresponding bucket comprising the lactate α-CH group (4.12 ppm) while there was no covariance for the remaining lipid fatty acyl chain resonances (Fig 6). Among the choline containing compounds, PC was increased upon drug treatment. At prolonged incubation time of 72 h, no significant changes in any of the lipid or choline (Cho, PC, GPC) signals contributed to distinguishing control and drug treated A2780cisR cells.

In HEK-293 cells, there was again a strong contribution from all lipid resonances in separating control and drug-treated cells as observed in A2780 cancer cells. However, the effects were opposite with increased lipid levels in response to 24 h drug treatment and a subsequent decrease after 72 h. PC and Cho were decreased after 24 h, while PC increased after 72 h.

**Fig 9:**
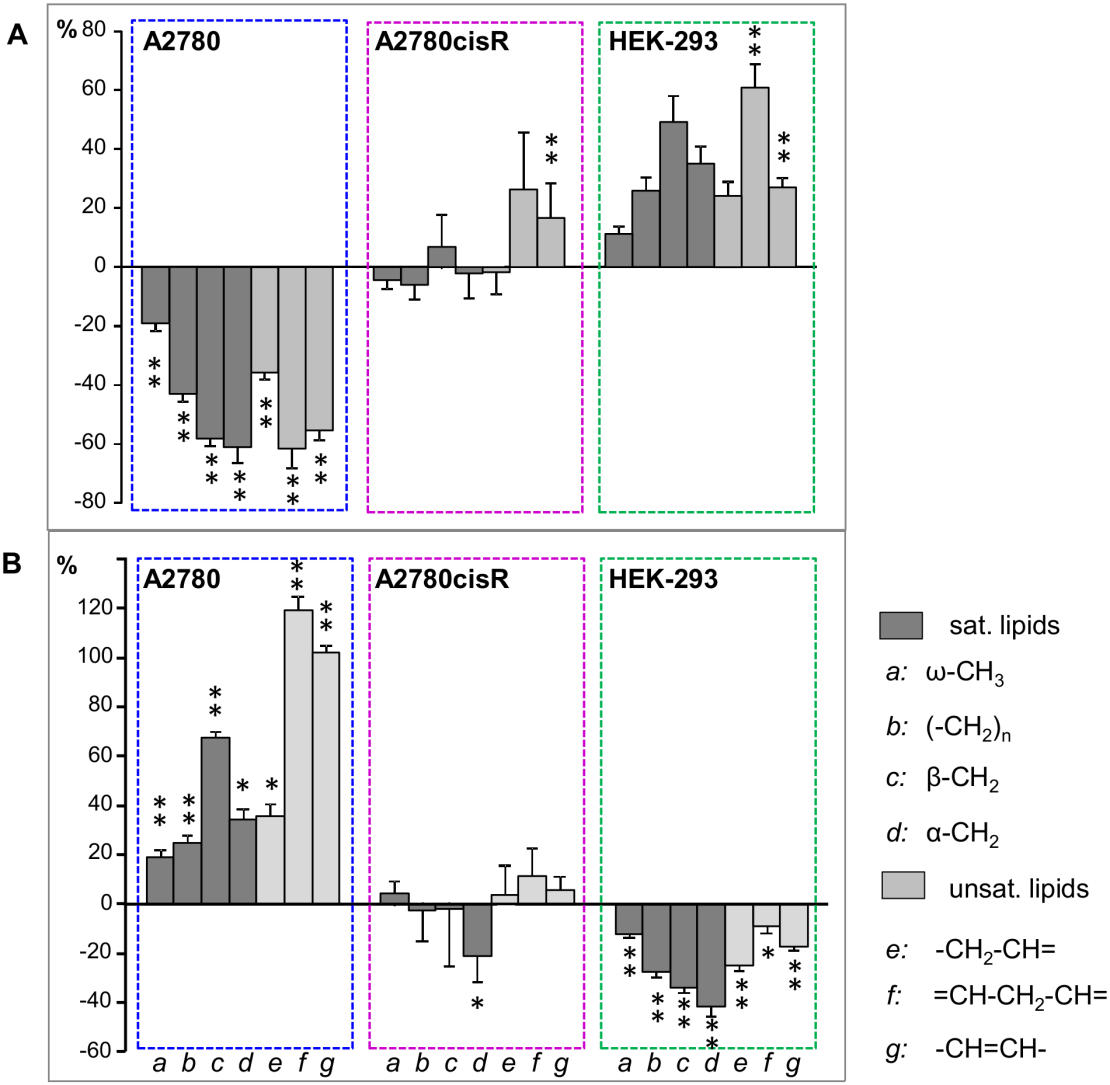
Relative changes for lipids. Relative differences (control—drug) of bucket integrals (means, PQN-normalized) with SE for saturated and unsaturated lipid resonances. (**A**) 24h and (**B**) 72h incubation time. * *p*<0.05; ** *p*<0.01 (corrected for multiple comparisons).

**Fig 10:**
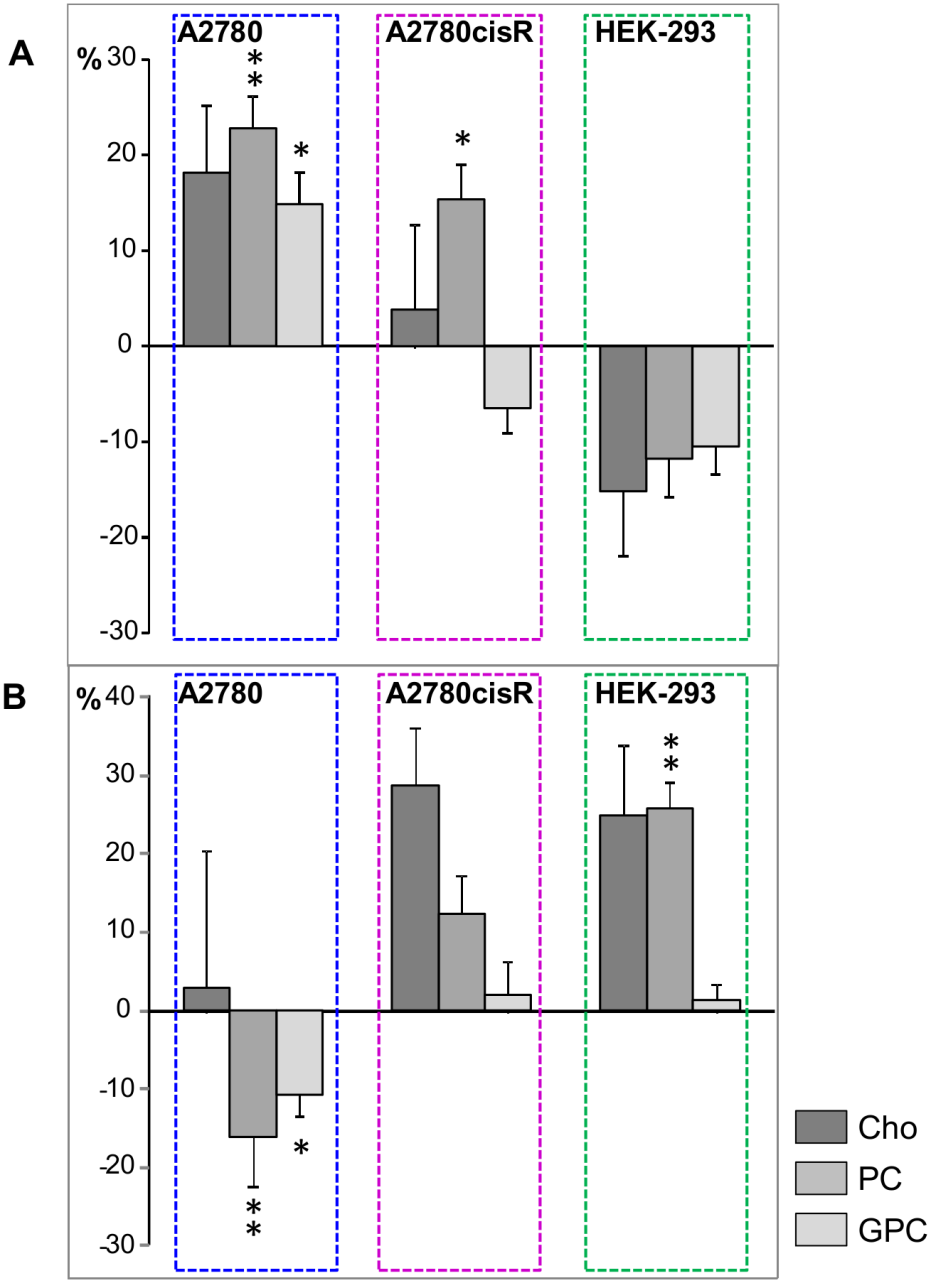
Relative changes for choline containing compounds. Relative differences (control—drug) of bucket integrals (means, PQN-normalized) with SE for choline containing compound resonances. (**A**) 24h and (**B**) 72h incubation time. * *p*<0.05; ** *p*<0.01 (corrected for multiple comparisons).

To compare the actual magnitude of alterations in lipids and choline-compounds induced by [**1**]^6+^ across the different cell lines and incubation periods, the relative differences of the normalized bucket means were plotted for the corresponding resonances (Figs 9 and 10). For the broad lipid—(CH_2_)_n_ resonance, which was split into 3 buckets (S7A Fig), only the upfield region not overlapping with lactate was evaluated. The plots in Figs 9 and 10 illustrate that in A2780 cells the cellular response was dominated by very large changes in the lipid resonances, which were initially all significantly reduced by up to 60% and subsequently all significantly increased, for unsaturated lipids even by more than 100% (Fig 9). Likewise, in HEK-293 cells induced differences in lipid signals were quite pronounced lying between 40 and 60% reduction after 72 h (p<0.05 for all) and increase after 24 h (p<0.01 for = CH-CH_2_-CH = and—CH = CH-), respectively, while the unsaturated lipid resonances in A2780cisR cells were only increased by about 20% (p<0.01 for—CH = CH- after 24 h). The observed changes in choline containing compounds were approximately on the same order of magnitude for the three cell lines with a significant increase (after 24 h) and subsequent decrease (after 72 h) for PC and GPC in A2780 cells, while in A2780cisR and HEK-293 cells, only PC exhibited a significant increase after 24 h and 72 h, respectively (Fig 10).

#### Changes in non-lipid small metabolites

**Fig 11:**
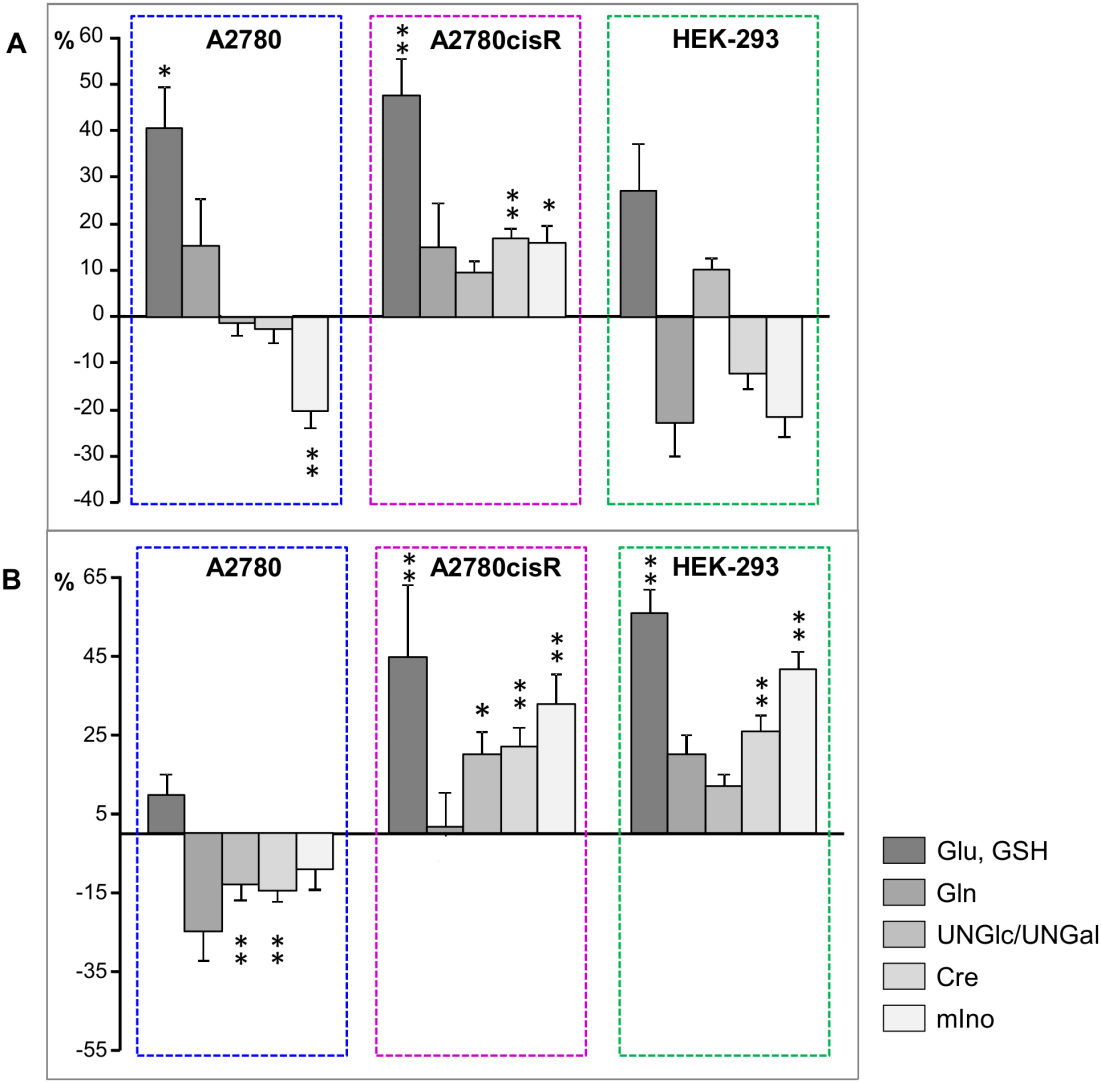
Relative changes for selected metabolites. Relative differences (control—drug) of bucket integrals (means, PQN-normalized) with SE for selected metabolite resonances. (**A**) 24h and (**B**) 72h incubation time. * *p*<0.05; ** *p*<0.01 (corrected for multiple comparisons).

In Fig 11, the relative differences for selected normalized mean bucket integrals are shown representing metabolites which were important besides lipids and choline in classifying control or drug treated cells according to their PLS load values (Figs 6 and 7). While multiple resonances give rise to the selected metabolite spectra the most isolated, i.e. least overlapping signal of each compound in the aliphatic region was chosen for integration (GSH/Glu: 2.57 ppm, Gln: 2.37 ppm, acetyl-group of UNGal and UNGlc: 2.09 ppm, Cre: 3.05 ppm, mIno: 3.57 ppm). In both cancer cell lines A2780 and A2780cisR glutathione (GSH), which overlaps with glutamate (Glu), clearly contributed to discrimination between control and drug treated cells. The metallaprism induced significantly increased levels of GSH/Glu up to 50% after 24 h incubation (Fig 11A), while in HEK-293 cells a similar increase was reached after 72 h (Fig 11B). Further significant contributions specifically in the cellular response of A2780cisR cells derived from N-acetylated nucleotide sugars (UNGal, UNGlc, after 72 h), creatine (Cre) and myo-inositol (m-Ino), which exhibited all increased levels both, after 24 h and 72 h (Fig 11). Similar alterations could be found in HEK-293 cells after 72 h drug incubation.

Additional metabolites contributing to the classification of drug treatment were found among the amino acids like lysine (Lys) and alanine (Ala) with increased levels in A2780 cells after 24 h (Fig 6) and decreased levels after 72 h (Fig 7). Furthermore, cholesterol compounds with a characteristic signal at 0.72 ppm and little overlap with other metabolites seem to play a role in both A2780 cancer cell lines responding to drug treatment (Figs 6 and 7).

### Cellular response to drug treatment—Analysis of single components in the spectral region 5–9 ppm

**Fig 12:**
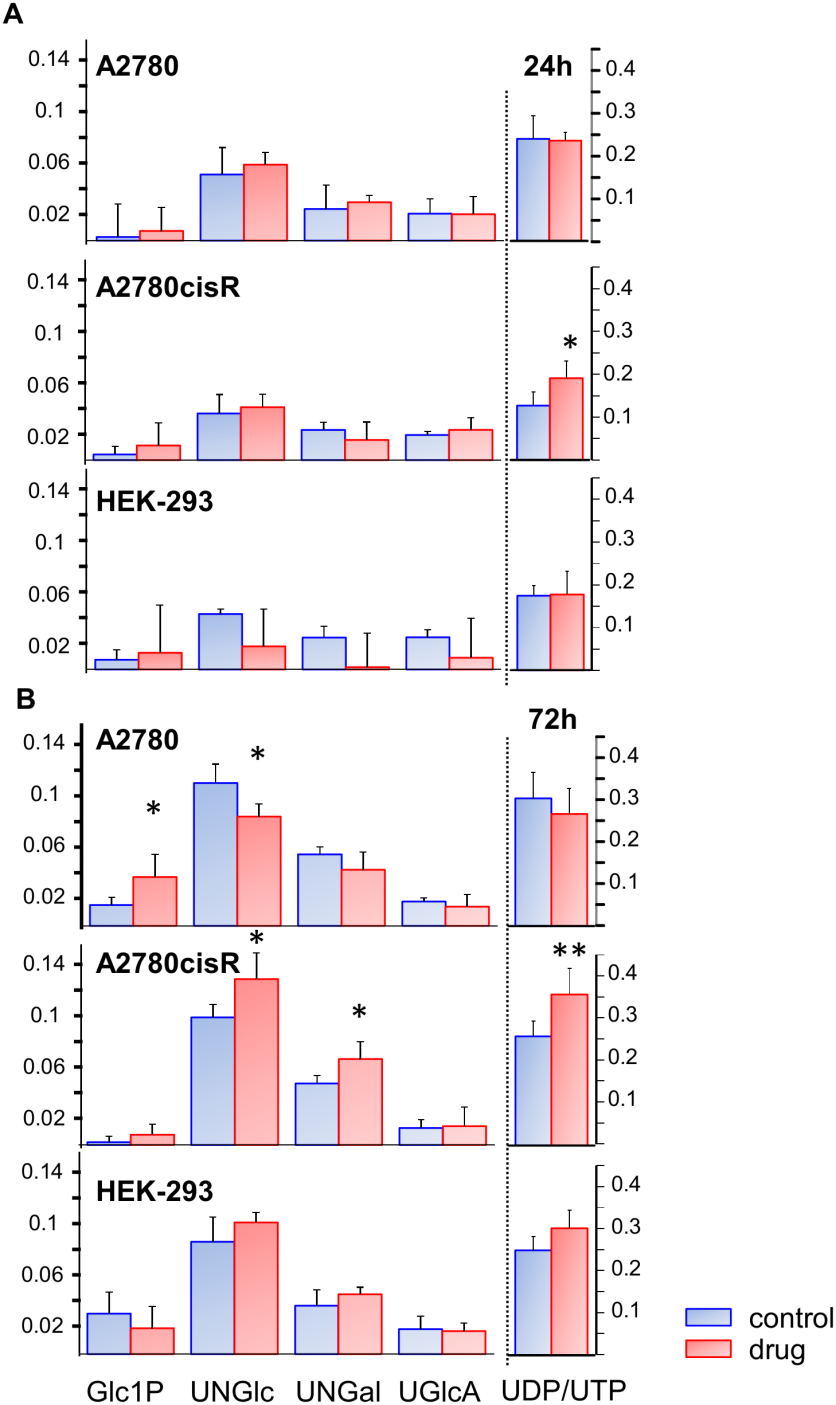
Integrals of metabolites from spectral region 5–9 ppm. Normalized mean integrals (± SD) of selected spectral regions from control and drug-treated A2780, A2780cisR and HEK-293 cell spectra (cpmg): α-Glucose-1-phosphate (Glc1P), UDP-N-acetylglucosamine (UNGlc), UDP-N-acetylgalactosamine (UNGal), UDP-glucuronic acid (UGlcA), and UDP/UTP. **(A)**: 24h, **(B)**: 72h incubation time. * *p*<0.05; ** *p*<0.01 (corrected for multiple comparisons).

Analysis of the spectral region between 5 and 9 ppm was performed on the *cpmg* spectra. In this region there is less overlap and the number of metabolite resonances is much smaller as compared to the aliphatic region (Fig 2). Therefore, single normalized integrals assigned to 11 different metabolites comprising various nucleotide derivatives and aromatic acids (Fig 2B) were evaluated in this region and tested for significant differences between control and drug-treated samples (S1 Table). Significant changes were found for several phosphate sugars and UDP/UTP in both cancer cell lines following 72 h drug incubation, as is shown in Fig 12. In particular, A2780cisR cells exhibited increased UDP/UTP levels after 24 h and more pronounced after 72 h. Simultaneously the two nucleotide sugars UDP-N-acetyl-glucosamine (UNGlc) and—galactosamine (UNGal) were significantly enhanced at 72 h. On the contrary, both sugars UNGlc and UNGal were reduced in A2780 cells while glucose-1-phosphate (Glc1P) was increased (Fig 12B). Both, the increase of UNGlc and UNGal in A2780cisR cells and a corresponding decrease in A2780 cells are in agreement with the results found for the N-acetyl group in the aliphatic spectral region (Fig 11).

For the remaining metabolites in the spectral region between 5 and 9 ppm, no significant changes could be observed except for the two amino acids tyrosine (Tyr) and phenylalanine (Phe), which were increased in A2780 cells and HEK-293 cells (only Tyr) at 72 h drug incubation (S1 Table).

### Potential processes underlying the different metabolic responses to Ru-complex treatment

The metabolic response to treatment with [**1**]^6+^ was not found to be uniform for the three different cell lines but seemed rather specific for each cell line. Moreover, the time course of the metabolite changes has to be considered since observed changes did not simply correlate with incubation time but were in part even opposite after prolonged periods of 72 h. In Table 3 the cellular compounds, which were clearly altered and thus important in distinguishing control and drug treated samples are summarized for each cell line and incubation time.

**Table 3:** Summary of cell compounds with increased (↑) or decreased (↓) levels in response to treatment with [1]^6+^.

Compound	A2780		A2780cisR		HEK-293	
	24h	72h	24h	72h	24h	72h
***Lipids***						
Lip ω-CH_3_	**↓**	**↑**			**↑**	**↓**
Lip (-CH_2_)_n_	**↓**	**↑**			**↑**	**↓**
Lip β-CH_2_	**↓**	**↑**			**↑**	**↓**
Lip-CH_2_-CH =	**↓**	**↑**			**↑**	**↓**
Lip α-CH_2_	**↓**	**↑**		**↓**	**↑**	**↓**
Lip = CH-CH_2_-CH =	**↓**	**↑**	**↑**		**↑**	
Lip-CH = CH-	**↓**	**↑**	**↑**		**↑**	**↓**
***Choline cont*. *cpds*.**						
Choline	**↑↓**	**↑**	**↑**	**↑**	**↓**	**↑**
PC	**↑**	**↓**	**↑**		**↓**	**↑**
GPC	**↑**	**↓**	**↓**			
***Phosphate-sugar/nuc*.**						
Glc-1P		**↑**				
UNGlc		**↓**		**↑**		
UNGal				**↑**		
UDP/UTP			**↑**	**↑**		
***Other metabolites***						
PE	**↓**		**↓**		**↑**	**↑**
Cholest. esters	**↓**	**↑**	**↑**	**↑**		
Glu, GSH	**↑**		**↑**	**↑**		**↑**
Gln			**↑**		**↓**	
Cre		**↓**	**↑**	**↑**	**↓**	**↑**
m-Ino	**↓**		**↑**	**↑**	**↓**	**↑**
Lac	**↓**		**↓**			**↓**
Lys	**↑**	**↓**			**↓**	
Ala	**↑**	**↓**	**↓**	**↓**		

#### Lipids and choline containing compounds

The meaning of altered bucket integrals can be twofold: Quantitative signal intensity alterations of metabolite resonances can be either due to a change in metabolite concentration or can reflect a change in mobility of the metabolite due to an altered chemical environment. Therefore, increased lipid signals may either indicate increased biosynthesis of mobile lipids or fatty acids, or enhanced membrane turnover [44] or degradation [35].

An increase of lipid signals, in particular of the methylene- and the terminal methyl-group has been reported as a typical marker for apoptosis where the biogenesis of cytoplasmic lipid droplets generally is enhanced [55]. In HEK-293 cells following 24 h drug incubation an overall trend towards increased lipids, significant for the unsaturated lipid resonances after Bonferroni correction, could be observed possibly reflecting an early stage of apoptosis. In contrast, membrane degradation seems rather unlikely to occur in HEK-293 cells since this would be accompanied by a simultaneous increase of PC and GPC as membrane breakdown products [35]. However, PC and GPC were found to be not significantly changed or rather decreased in HEK-293 cells after 24 h. Similarly, increased lipid signals have also been found in several studies in response to drug treatment and were related to a rise in lipid droplets [30,56–58]. The reported simultaneous drop of PC accompanying the increase of mobile lipids is also in accordance with our findings. The inversion of the lipid changes towards a relative decrease, which however was less pronounced but significant for all lipid signals at prolonged incubation time (Fig 9), may be explained by the different ground level state of the control cells (Fig 3). Comparison of HEK-293 cells grown for 24 h and 72 h indicated that the control cells already acquired increased lipid levels after 72 h (S13 Fig), which may be due to cell stress during the advanced growth phase [53]. The observed decrease upon 72 h drug treatment may therefore either be caused by the drug slowing down the lipid production or, more likely may reflect an advanced stage of apoptotic cell death pathway with consumption of lipids as energy stores [58]. Leaky plasma cell membranes with enhanced excretion of intracellular lipids into the culture medium may also cause decreased lipids. Since the culture medium is replaced before cell measurements, excreted compounds would not contribute to the cell spectra but would result in decreased levels of the corresponding metabolites. This however seems rather unlikely since membrane integrity was found to be maintained according to Trypan blue assays performed before HR-MAS NMR measurements. Further studies such as microscopy would be required to clarify the causes for the NMR results.

Compared to HEK-293 cells, the response after 24 h in A2780 cancer cells was contrary with respect to the lipid signals: here, a pronounced and significant decrease of all lipid resonances was observed accompanied with a strong increase of choline containing compounds. This suggests that the metallaprism triggers different mechanisms in A2780 cells. A relative increase of all metabolites including choline with respect to lipids has been reported to indicate cell death pathway *via* necrosis [59]. On the other hand, an overall reduction of most metabolites including lipids has also been reported to be associated with cellular necrotic response [44]. A typical feature of necrosis is the loss of membrane integrity [60]. This may result in enhanced release of small metabolites into the cytosol and might thus account for a relative decrease of lipids. The subsequent increase of all lipid resonances at 72h, in particular the unsaturated lipids, may be a sign of enhanced beta-oxidation and membrane breakdown releasing fatty acids.

Although the interpretation of the results is speculative, the converse effects found for the metabolic responses in HEK-293 cells and A2780 cells depending on the incubation time demonstrate that the choice of time point during the growth phase for metabolic profiling is an important factor to be considered when interpreting the results since different apoptotic stages or alternatively stages towards necrosis evolve over time [55,61].

In A2780cisR cells the overall lipid content remained more or less unchanged while there was an increase in the unsaturated lipid signals (significant for—CH = CH-) upon treatment with [**1**]^6+^. This indicates that the lipid biosynthesis may not be enhanced but rather that existing lipids undergo transformation. Interestingly, a similar selective increase of poly-unsaturated fatty acids (PUFAs) with no concomitant increase of total lipids has been recently reported for osteosarcoma cells responding to doxorubicin and methotrexate treatment [32]. Moreover, an increase particularly of unsaturated lipids has been reported for cancer cells responding to cisplatin treatment [28] or to other treatment modalities [62] and was interpreted as the onset of apoptosis. In the latter study a simultaneous increase of cholesteryl esters was found, which was also observed in A2780cisR cells after 24 h and 72 h as indicated by an increase in the resonance at 0.72 ppm (Figs 6 and 7, Table 3) suggesting the formation of cholesteryl esters with unsaturated fatty acids. The increase of PUFAs has been ascribed to their liberation from membranes through phospholipase A2 activity [62] also leading to increase of PC which is in agreement with our findings. However, an increase of PUFAs has been found to depend on the cell type and growth conditions and is not always observed in apoptosis [58].

#### Nucleotides and nucleotide sugars

The results obtained from the downfield spectral region (Fig 12) support the findings related to the lipid signals. After 72 h drug incubation, the nucleotide compounds (UDP/UTP, UNGlc and UNGal) were significantly increased in A2780cisR cells, increased in HEK-293 cells and reduced in A2780 cells. Nucleotide sugars serve as sugar donors in glycosylation processes such as the biosynthesis of glycoproteins or glycogen [63,64]. Their increase suggests reduced protein and lipid glycosylation or reduced enzyme activity for this step. Similar to our finding for A2780cisR cells responding to ruthenium complex treatment, a correlated increase of unsaturated lipids and nucleotide sugars has also been reported for cisplatin sensitive cells [28,30] but was lacking in cisplatin resistant cells [30]. UNGlc and UNGal were suggested as early markers for cisplatin treatment response [30], however, an increase seems to depend on the cell type, since it was not observed in cisplatin treated osteosarcoma cells [29]. The similarity in the response triggered by the metallaprism in cisplatin resistant A2780cisR cells suggests that similar biological processes seem to be involved while the resistance mechanism seems to be deactivated by the ruthenium complex. The response in cisplatin sensitive A2780 cells on the other hand seems to follow different pathways, possibly of necrotic type, since here rather a decrease in nucleotides, significant for UNGlc, was observed. A general increase of high energy phosphates like UDP and UTP has been reported to be typical for the early stage of apoptosis where still high levels of such compounds are maintained, whereas their decrease rather indicates necrotic cell death [55]. Nevertheless, elevated levels of nucleotide sugars in the absence of drug were observed in all three cell lines at prolonged growth times of 72 h as compared to 24 h indicating that the increase is not specific for drug-induced apoptosis.

#### Glutamate and glutathione

An interesting observation was the increase in glutamate (Glu) and glutathione (GSH) found throughout in all cell lines both at short and long incubation times (Fig 11). In most studies reported in the literature, reduced levels of GSH have been found in cells responding to different antitumor drugs [29,34,59,65] and have been related to oxidative stress. An important role of GSH is the detoxification of reactive oxygen species (ROS) catalyzed by GSH S-transferase (GST) [48]. Overexpression of GST has been found to correlate with drug resistance in cisplatin resistant ovarian cancer cells [66] and cisplatin as well as carboplatin and oxaliplatin were reported to form adducts with GSH in vitro possibly reducing their cytotoxic activity [67]. In our group no adduct formation was observed between the metallaprism [**1**]^6+^ and GSH, however, [**1**]^6+^ catalyzed the oxidation of GSH [14]. The increased GSH levels observed in all cell lines following treatment with [**1**]^6+^ most likely reflect enhanced GSH production to induce the detoxification. However, this cellular defense mechanism seems not to be effective against [**1**]^6+^. A possible reason for GSH accumulation may be that the GST catalyzed oxidation is inhibited by the metallaprism. Dose-dependent GST inhibition by polyphenolic compounds leading to increased intracellular GSH levels has been previously reported [68]. The dhnq ligand of [**1**]^6+^ (Fig 1) may play a role as potential GST inhibitor since naphthoquinones have been reported to inhibit GST activity in-vitro [69,70]. Further investigations will be required to test this hypothesis.

## Conclusions

In conclusion, the presented HR-MAS NMR based metabonomic data revealed different metabolic responses towards treatment under same conditions with the hexacationic ruthenium metallaprism [**1**]^6+^ depending on the cell line and the incubation time. The cell-type specific responses suggest different possible pathways of cell death induced by [**1**]^6+^. Pronounced effects induced on A2780 cancer cells may be caused by early necrotic stages while the cisplatin resistant A2780cisR and HEK-293 cells more likely seem to follow apoptotic cell death pathways. Metabolic profiles of untreated cells underwent time-dependent changes representing different stages of cellular growth in culture. Thus, HR-MAS NMR presents a snapshot of the metabolic state of the cells. Against this background metabolic transitions from short to long incubation times with [**1**]^6+^ observed for HEK-293 and cisplatin resistant A2780cisR cells on one side, and for A2780 cells on the other side, may reflect different stages towards their suggested cell death pathways.

Analysis of the most pronounced chemical alterations provides indications of possible mechanistic processes involved in the cytotoxic effects of [**1**]^6+^. In A2780 and HEK-293 cells mainly the lipid metabolism was affected, however, with changes in opposite directions. For cisplatin resistant A2780 cells increased nucleotide sugars and unsaturated lipid components suggest that lipid biogenesis and glycosylation pathways are involved in the treatment response. Increased GSH levels found in all responding cell lines suggested the potential of [**1**]^6+^ to bypass cellular detoxification processes, which may be part of the cytotoxic active principle of [**1**]^6+^.

Nevertheless, the suggested cell death pathways and metabolic processes involved remain hypothetical and additional investigations including cell morphological studies will be required to further support the conclusions drawn from the present study. Resonance signals which proved to be sensitive towards [**1**]^6+^ may serve as biomarkers for treatment response and may be helpful for monitoring future studies aimed at the design of more selective Ru complexes as potential new alternative anti-cancer drugs.

## Supporting Information

S1 Fig HR-MAS ^1^H-spectra of fresh and thawed cells. HR-MAS ^1^H-spectra comparing freshly harvested and thawed cell suspensions for A2780, A2780cisR, and HEK-293 cell suspension in PBS, spectral region 0.5–4.6 ppm.
(TIF)

S2 Fig Time-dependent HR-MAS ^1^H spectra of a cell suspension. HR-MAS ^1^H spectra for a A2780cisR cell suspension in PBS recorded directly (0h), after 3h, 6h, 9h, and 13h under MAS conditions (MAS 3kHz) and T = 37°C. Spectral region 0.5–4.7 ppm and expansions.
(TIF)

S3 Fig HR-MAS ^1^H^1^H-TOCSY (0.5–5.5ppm). HR-MAS ^1^H^1^H-TOCSY spectrum for a A2780 cell suspension in PBS, spectral region 0.5–5.5 ppm.
(TIF)

S4 Fig HR-MAS ^1^H^1^H-TOCSY (5.0–8.8ppm). HR-MAS ^1^H^1^H-TOCSY spectrum for a A2780 cell suspension in PBS, spectral region 5.0–8.8 ppm.
(TIF)

S5 Fig HR-MAS ^1^H-J-resolved (0.5–4.5 ppm). HR-MAS ^1^H-J-resolved spectrum for a A2780 cell suspension in PBS, spectral region 0.5–4.5 ppm.
(TIF)

S6 Fig HR-MAS ^1^H-J-resolved (5.0–8.8 ppm). HR-MAS ^1^H-J-resolved spectrum for a A2780 cell suspension in PBS, spectral region 5.0–8.8 ppm.
(TIF)

S7 Fig Bucket selection. Individually sized bucket selection shown on an overlay of spectra obtained for A2780cisR control & drug treated cells (72h). The filled regions were excluded due to contaminations from ethanol, DMSO, and proximity to the residual water signal. **(A)**: 0.5–2.5 ppm, (**B)**: 2.5–4.5 ppm, (**C)**: 4.9–6.5 ppm.
(TIF)

S8 Fig PCA of control and drug treated cells. PCA scores plots based on 1D noesy HR-MAS spectral regions between 0.5 and 6.5 ppm (97 buckets) of control (blue circles) and drug treated (green crosses) cells for A2780 (**A, B**), A2780cisR (**C, D**) and HEK-293 cells (**E, F**) at incubation times of 24h and 72h. Red: 95% confidence interval.
(TIF)

S9 Fig PLS of control cells grown for 24h. PLS scores plot based on 1D noesy HR-MAS spectral regions between 0.5 and 6.5 ppm (97 buckets) of control cells A2780, A2780cisR, and HEK-293 cells grown for 24h. Blue line: 95% confidence level.
(TIF)

S10 Fig Loading plots for LV1 and LV2. Loading plots for PLS scores shown in S9 Fig and 97 variables (= buckets): (**A**) Loadings on the first PLS component LV1 and (**B**) for the second PLS component LV2. For bucket assignments, see S2 Table. Annotated buckets in (A) derive from lipids and in (B) from lactate, uridine, and amino acids.
(TIF)

S11 Fig PLS of A2780 control cells at 24h and 72h. **(A)** PLS scores plot based on 1D noesy HR-MAS spectral regions between 0.5 and 6.5 ppm (97 buckets) of A2780 control cells grown for 24h (red) and 72h (blue). Blue line: 95% confidence level **(B)** Corresponding PLS loadings for LV-1. For bucket assignments, see S2 Table.
(TIF)

S12 Fig PLS of A2780cisR control cells at 24h and 72h. **(A)** PLS scores plot based on 1D noesy HR-MAS spectral regions between 0.5 and 6.5 ppm (97 buckets) of A2780cisR control cells grown for 24h (red) and 72h (blue). Blue line: 95% confidence level. **(B)** Corresponding PLS loadings for LV-1. For bucket assignments, see S2 Table.
(TIF)

S13 Fig PLS of HEK-293 control cells at 24h and 72h. **(A)** PLS scores plot based on 1D noesy HR-MAS spectral regions between 0.5 and 6.5 ppm (97 buckets) of HEK-293 control cells grown for 24h (red) and 72h (blue). Blue line: 95% confidence level. **(B)** Corresponding PLS loadings for LV-1. For bucket assignments, see S2 Table.
(TIF)

S14 Fig PLS loadings for A2780 ctrl vs drug. Loading plots for the first PLS component LV1 and 97 variables (= buckets) for PLS comparing A2780 control cells versus drug treated at **(A)** 24h and **(B)** 72h. Red line: arbitrary threshold set to a load value of +/- 0.1.
(TIF)

S15 Fig PLS loadings for A2780cisR ctrl vs drug. Loading plots for the first PLS component LV1 and 97 variables (= buckets) for PLS comparing A2780cisR control cells versus drug treated at **(A)** 24h and **(B)** 72h. Red line: arbitrary threshold set to a load value of +/- 0.1.
(TIF)

S16 Fig PLS loadings for HEK-293 ctrl vs drug. Loading plots for the first PLS component LV1 and 97 variables (= buckets) for PLS comparing HEK-293 control cells versus drug treated at **(A)** 24h and **(B)** 72h. Red line: arbitrary threshold set to a load value of +/- 0.1.
(TIF)

S1 Table Bucket integrals (± SD) and *p*-values for spectral region 5–9 ppm. (PDF)

S2 Table Assignment and spectral regions of buckets. (PDF)
