# Supplementary figures and images for: 1H HR-MAS NMR Based Metabolic Profiling of Cells in Response to Treatment with a Hexacationic Ruthenium Metallaprism as Potential Anticancer Drug
Source: PLoS One. 2015 May 29;10(5):e0128478. doi: 10.1371/journal.pone.0128478 (PMC4449131; doi:10.1371/journal.pone.0128478)

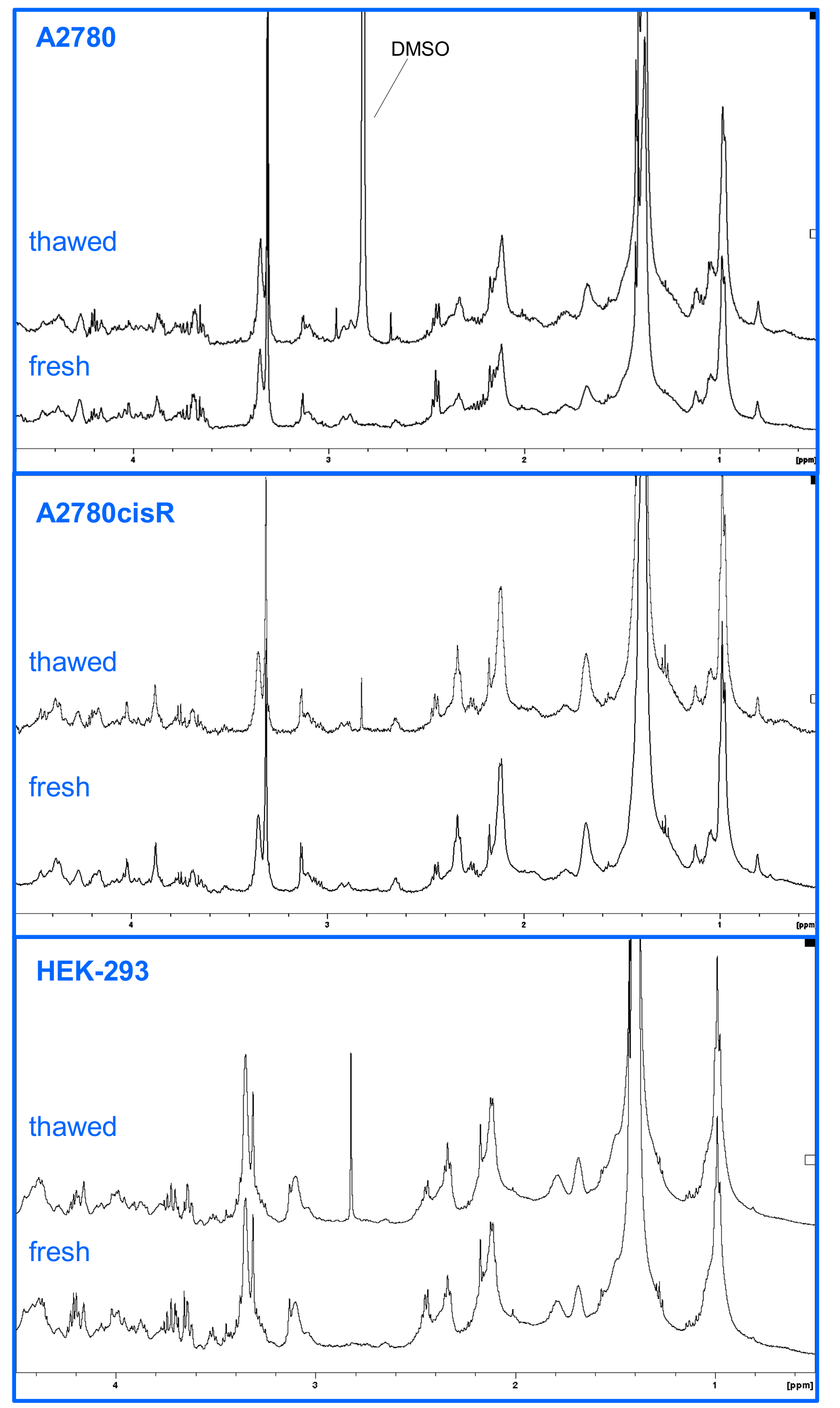

Supplement: S1 Fig — HR-MAS 1H-spectra comparing freshly harvested and thawed cell suspensions for A2780, A2780cisR, and HEK-293 cell suspension in PBS, spectral region 0.5–4.6 ppm. (TIF) [file pone.0128478.s001.tif]

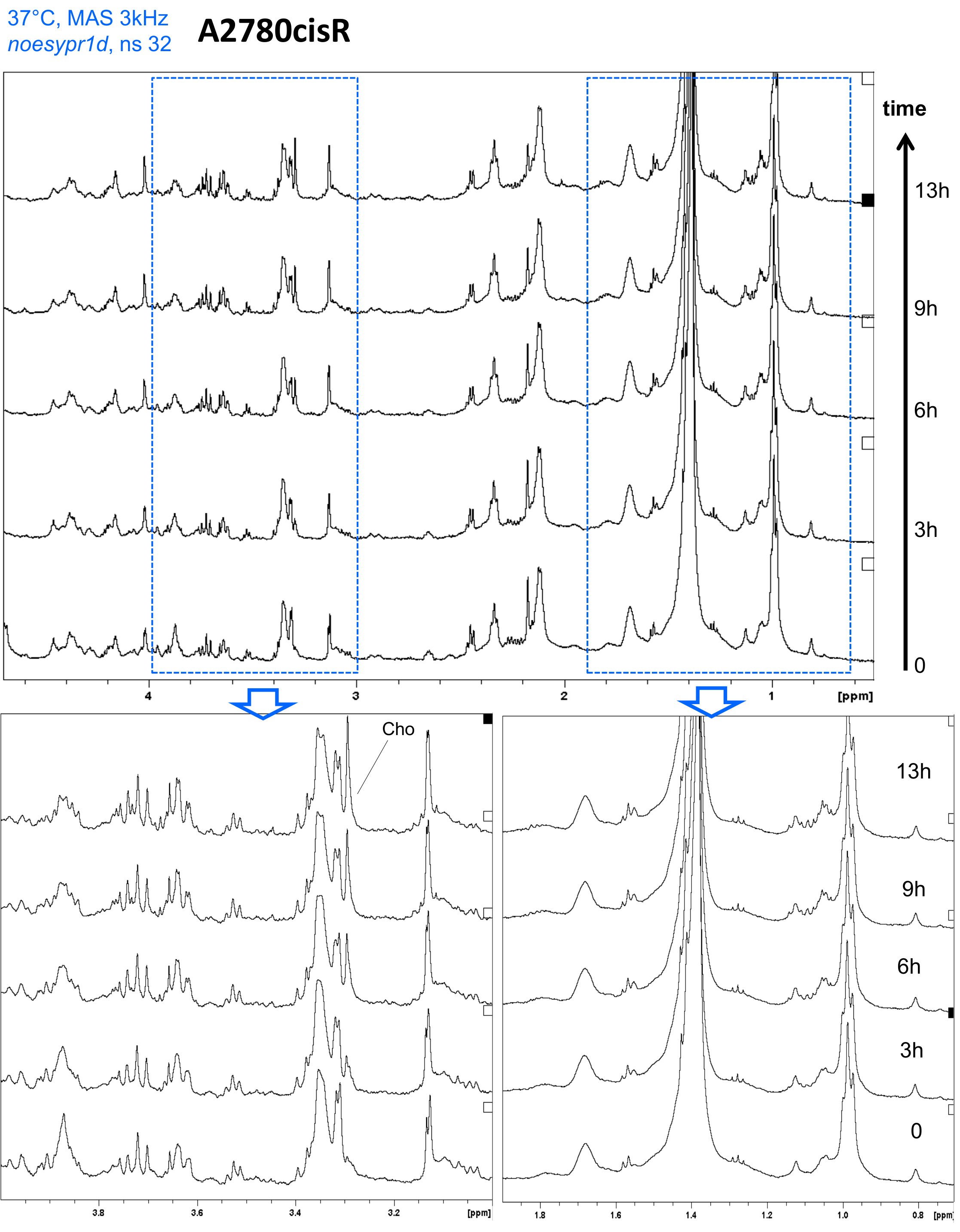

Supplement: S2 Fig — HR-MAS 1H spectra for a A2780cisR cell suspension in PBS recorded directly (0h), after 3h, 6h, 9h, and 13h under MAS conditions (MAS 3kHz) and T = 37°C. Spectral region 0.5–4.7 ppm and expansions. (TIF) [file pone.0128478.s002.tif]

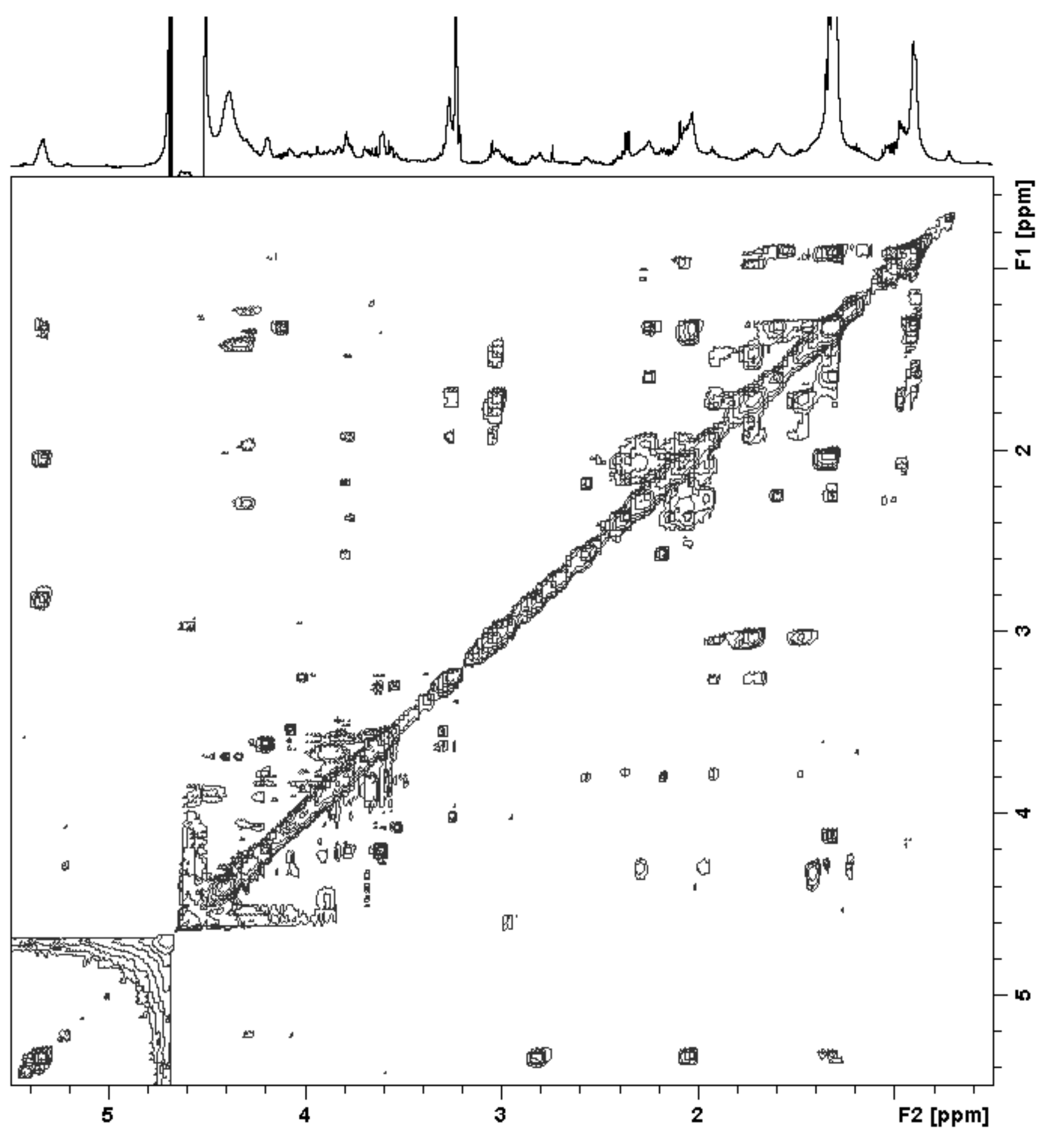

Supplement: S3 Fig — HR-MAS 1H1H-TOCSY spectrum for a A2780 cell suspension in PBS, spectral region 0.5–5.5 ppm. (TIF) [file pone.0128478.s003.tif]

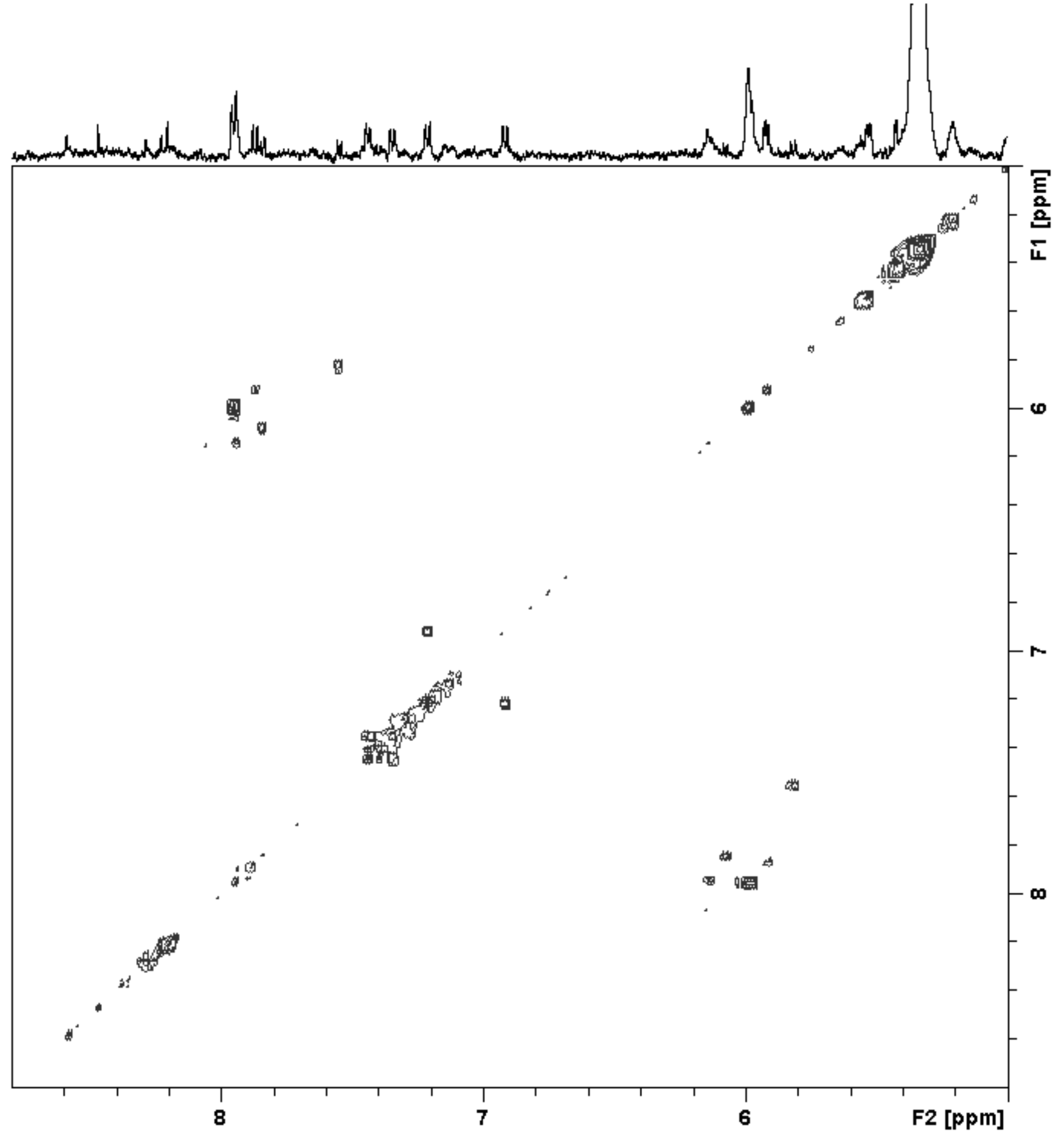

Supplement: S4 Fig — HR-MAS 1H1H-TOCSY spectrum for a A2780 cell suspension in PBS, spectral region 5.0–8.8 ppm. (TIF) [file pone.0128478.s004.tif]

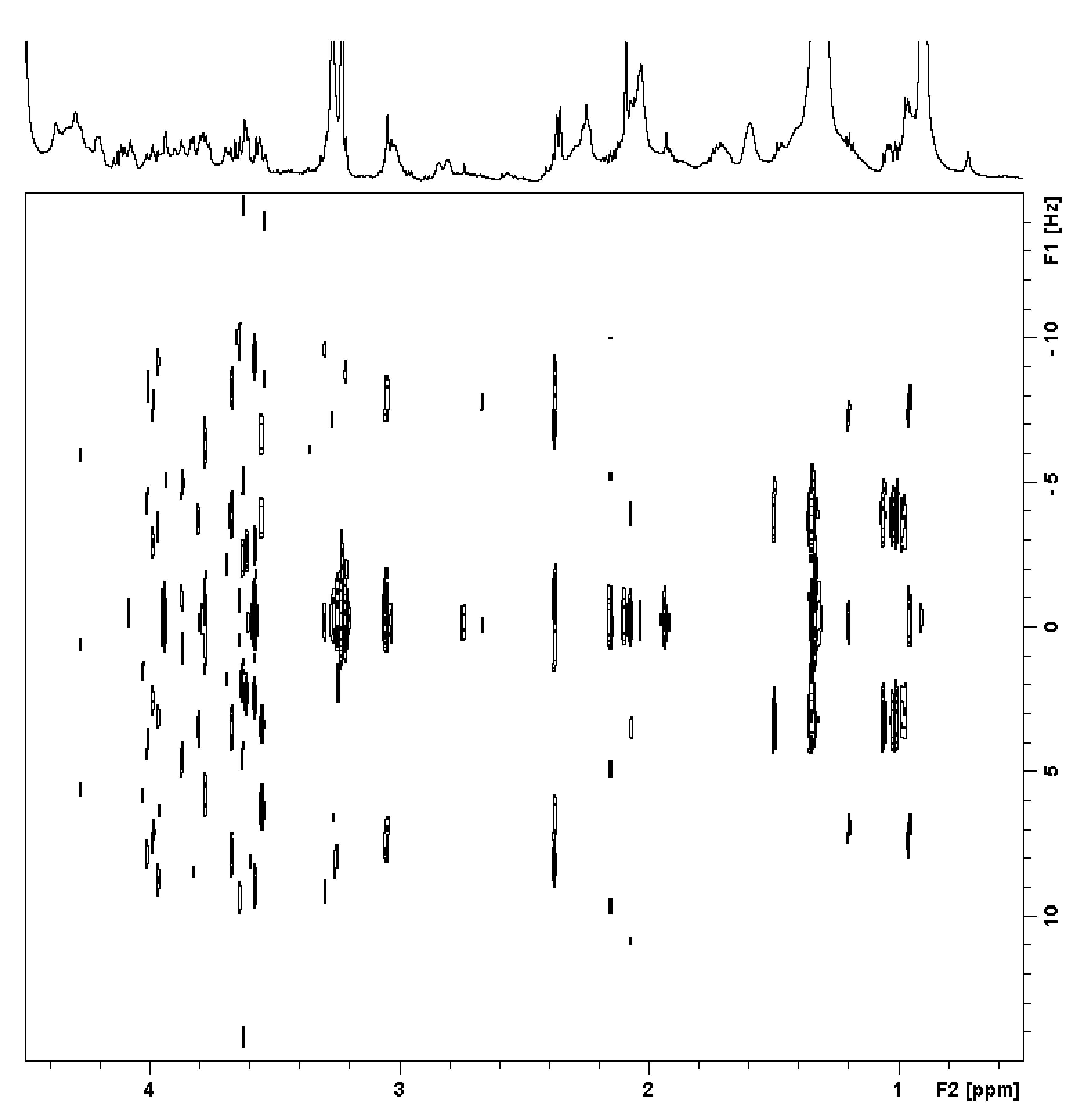

Supplement: S5 Fig — HR-MAS 1H-J-resolved spectrum for a A2780 cell suspension in PBS, spectral region 0.5–4.5 ppm. (TIF) [file pone.0128478.s005.tif]

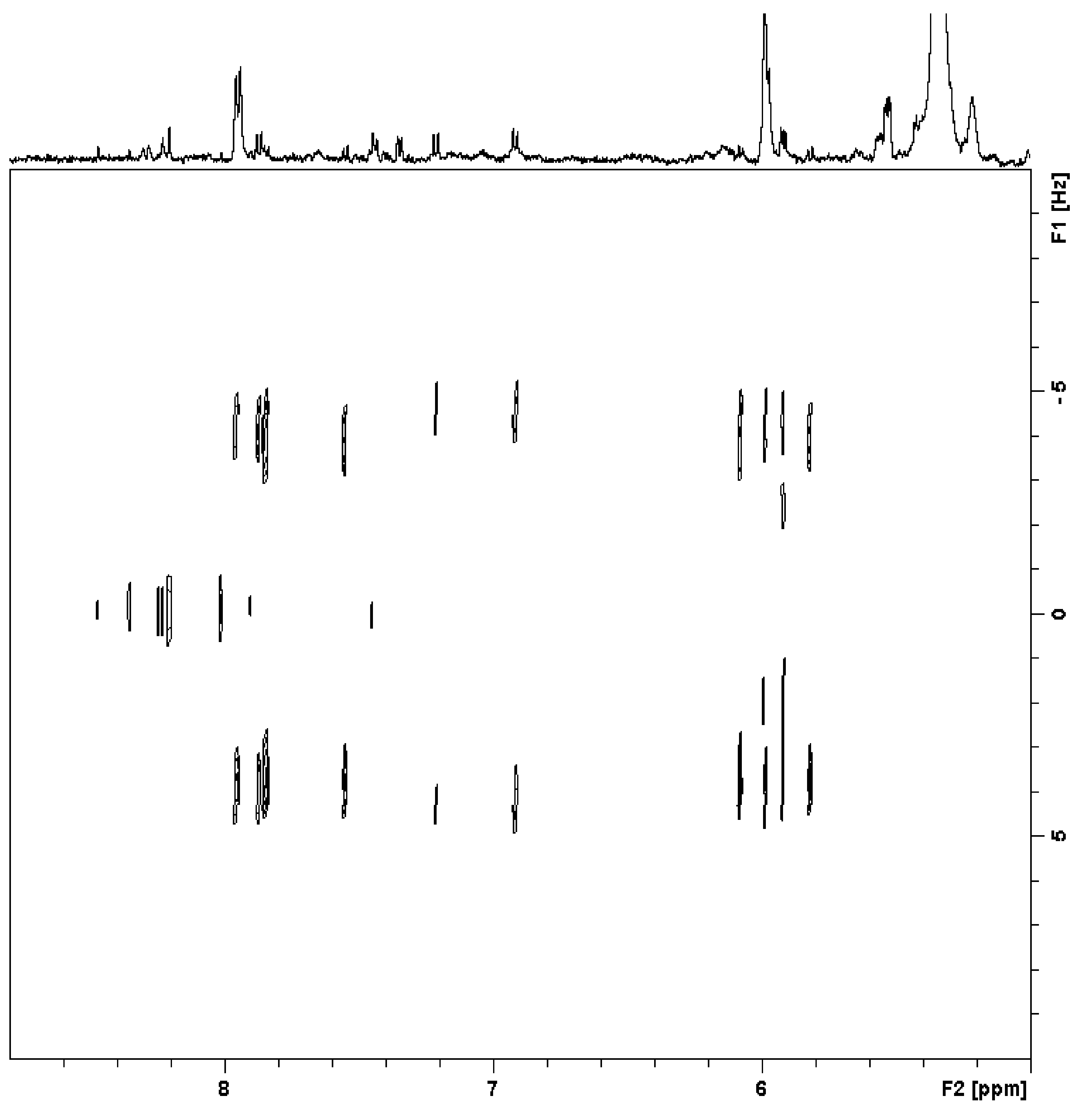

Supplement: S6 Fig — HR-MAS 1H-J-resolved spectrum for a A2780 cell suspension in PBS, spectral region 5.0–8.8 ppm. (TIF) [file pone.0128478.s006.tif]

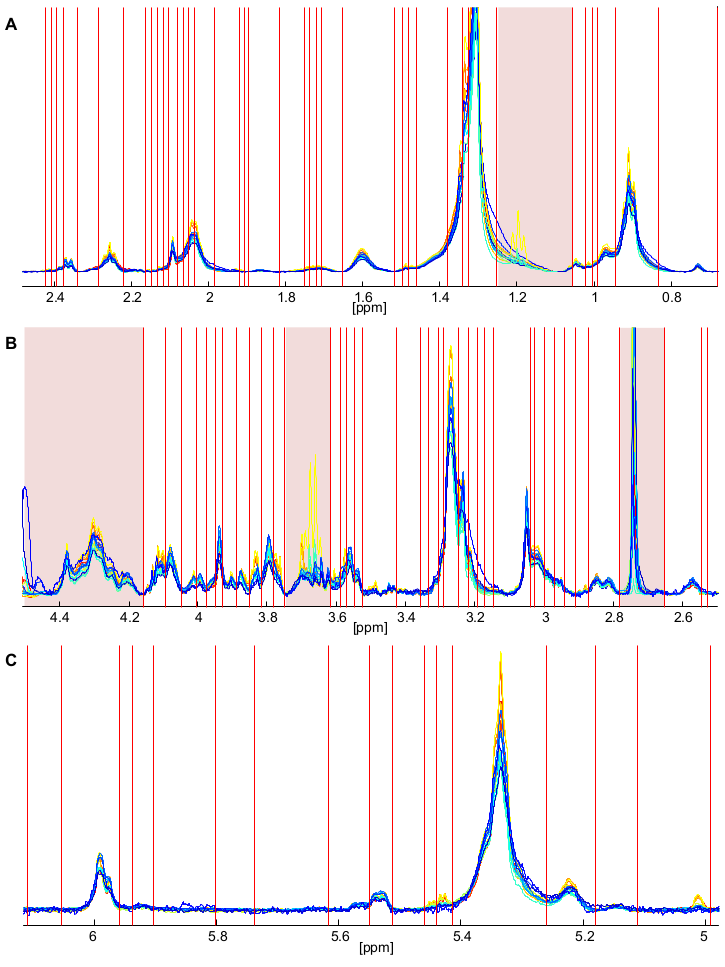

Supplement: S7 Fig — Individually sized bucket selection shown on an overlay of spectra obtained for A2780cisR control & drug treated cells (72h). The filled regions were excluded due to contaminations from ethanol, DMSO, and proximity to the residual water signal. (A): 0.5–2.5 ppm, (B): 2.5–4.5 ppm, (C): 4.9–6.5 ppm. (TIF) [file pone.0128478.s007.tif]

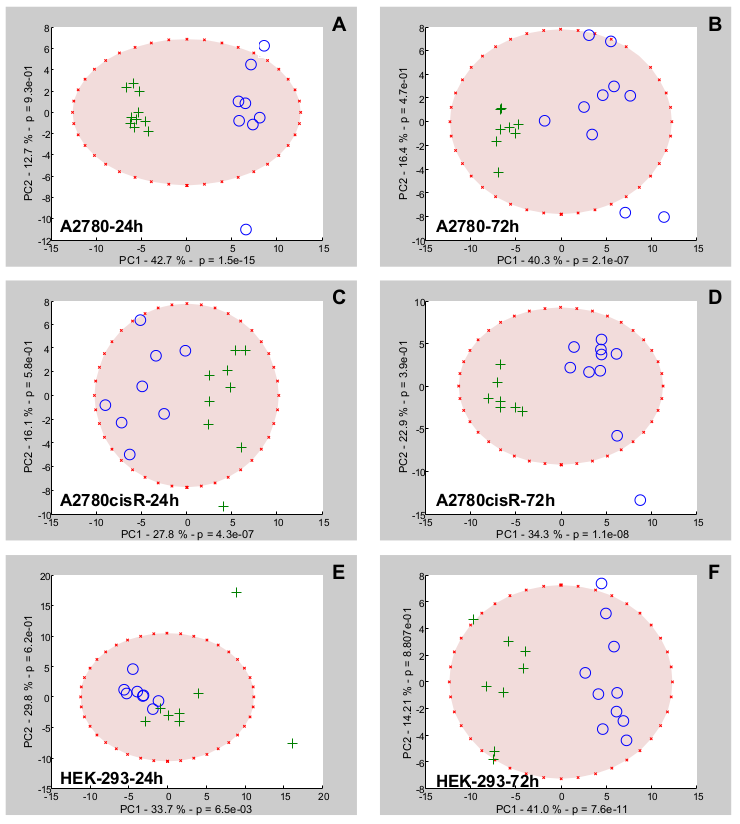

Supplement: S8 Fig — PCA scores plots based on 1D noesy HR-MAS spectral regions between 0.5 and 6.5 ppm (97 buckets) of control (blue circles) and drug treated (green crosses) cells for A2780 (A, B), A2780cisR (C, D) and HEK-293 cells (E, F) at incubation times of 24h and 72h. Red: 95% confidence interval. (TIF) [file pone.0128478.s008.tif]

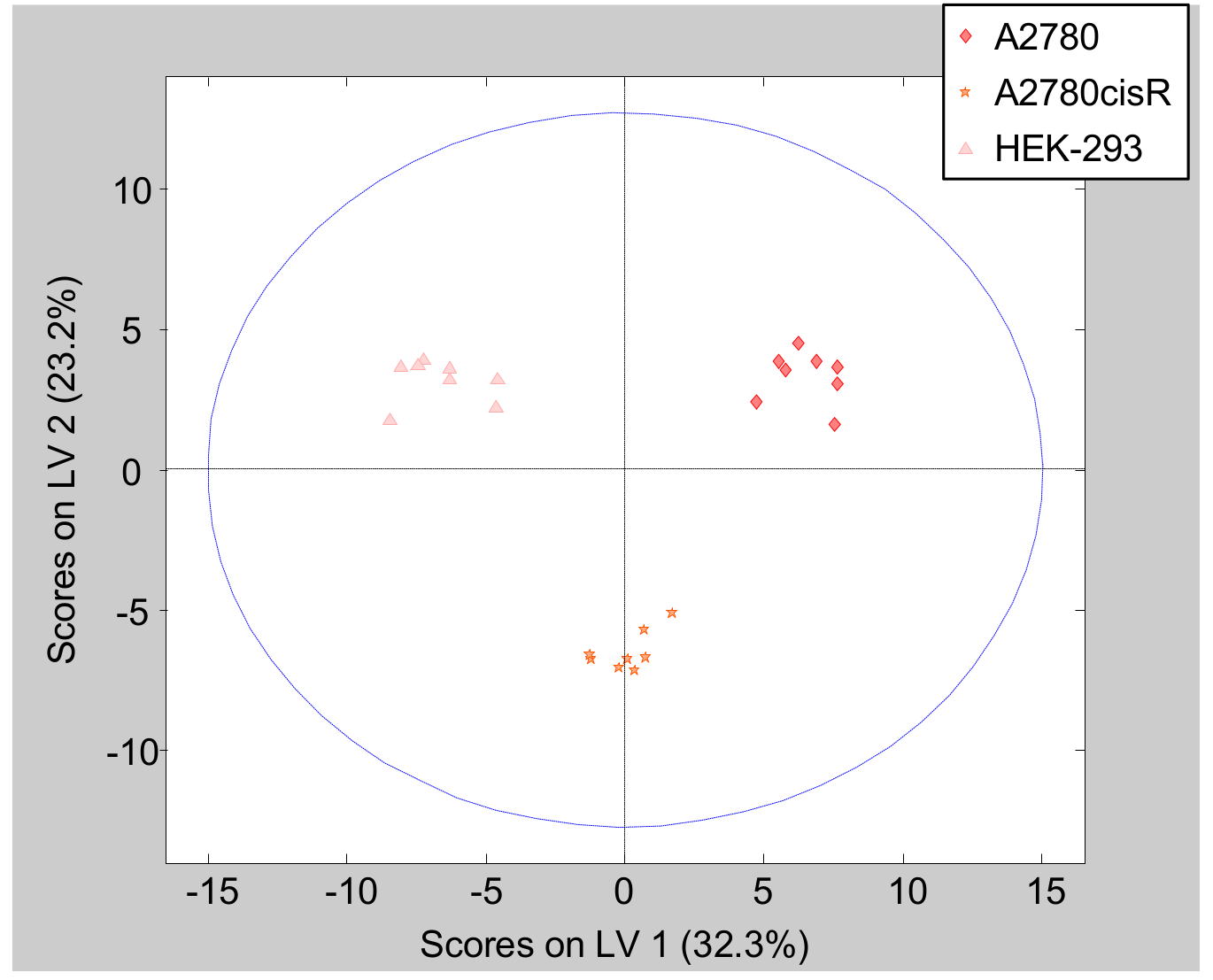

Supplement: S9 Fig — PLS scores plot based on 1D noesy HR-MAS spectral regions between 0.5 and 6.5 ppm (97 buckets) of control cells A2780, A2780cisR, and HEK-293 cells grown for 24h. Blue line: 95% confidence level. (TIF) [file pone.0128478.s009.tif]

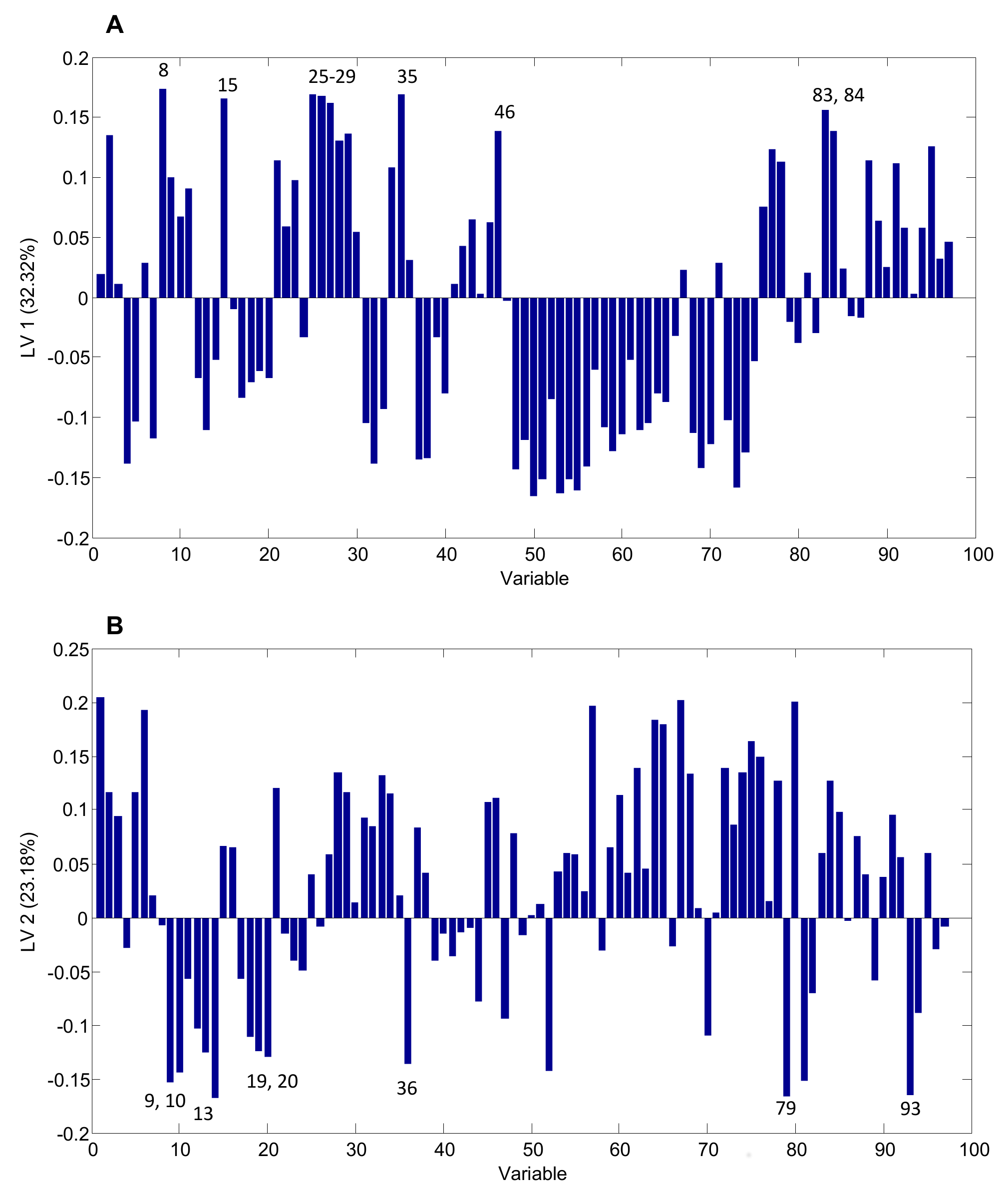

Supplement: S10 Fig — Loading plots for PLS scores shown in S9 Fig and 97 variables (= buckets): (A) Loadings on the first PLS component LV1 and (B) for the second PLS component LV2. For bucket assignments, see S2 Table. Annotated buckets in (A) derive from lipids and in (B) from lactate, uridine, and amino acids. (TIF) [file pone.0128478.s010.tif]

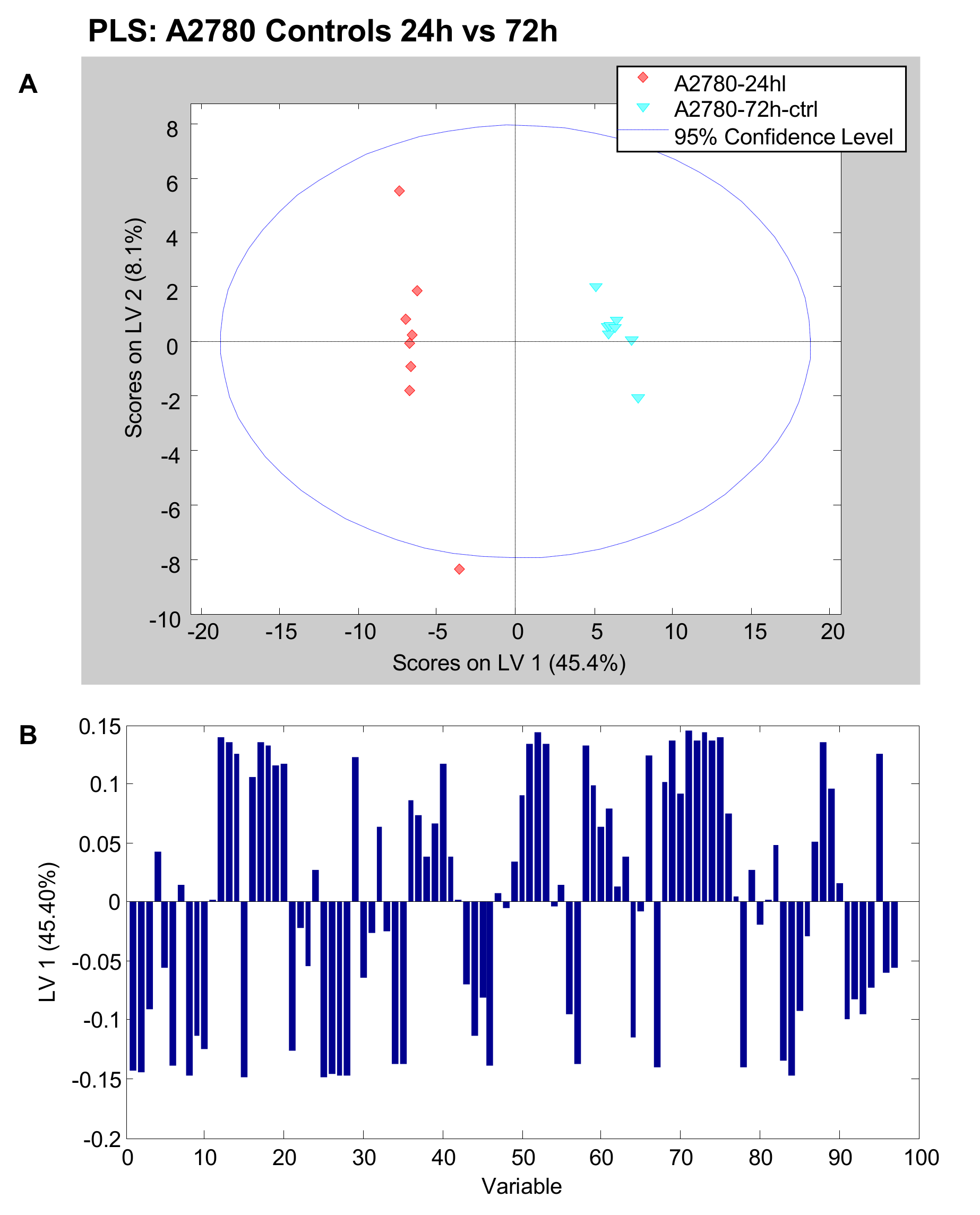

Supplement: S11 Fig — (A) PLS scores plot based on 1D noesy HR-MAS spectral regions between 0.5 and 6.5 ppm (97 buckets) of A2780 control cells grown for 24h (red) and 72h (blue). Blue line: 95% confidence level (B) Corresponding PLS loadings for LV-1. For bucket assignments, see S2 Table. (TIF) [file pone.0128478.s011.tif]

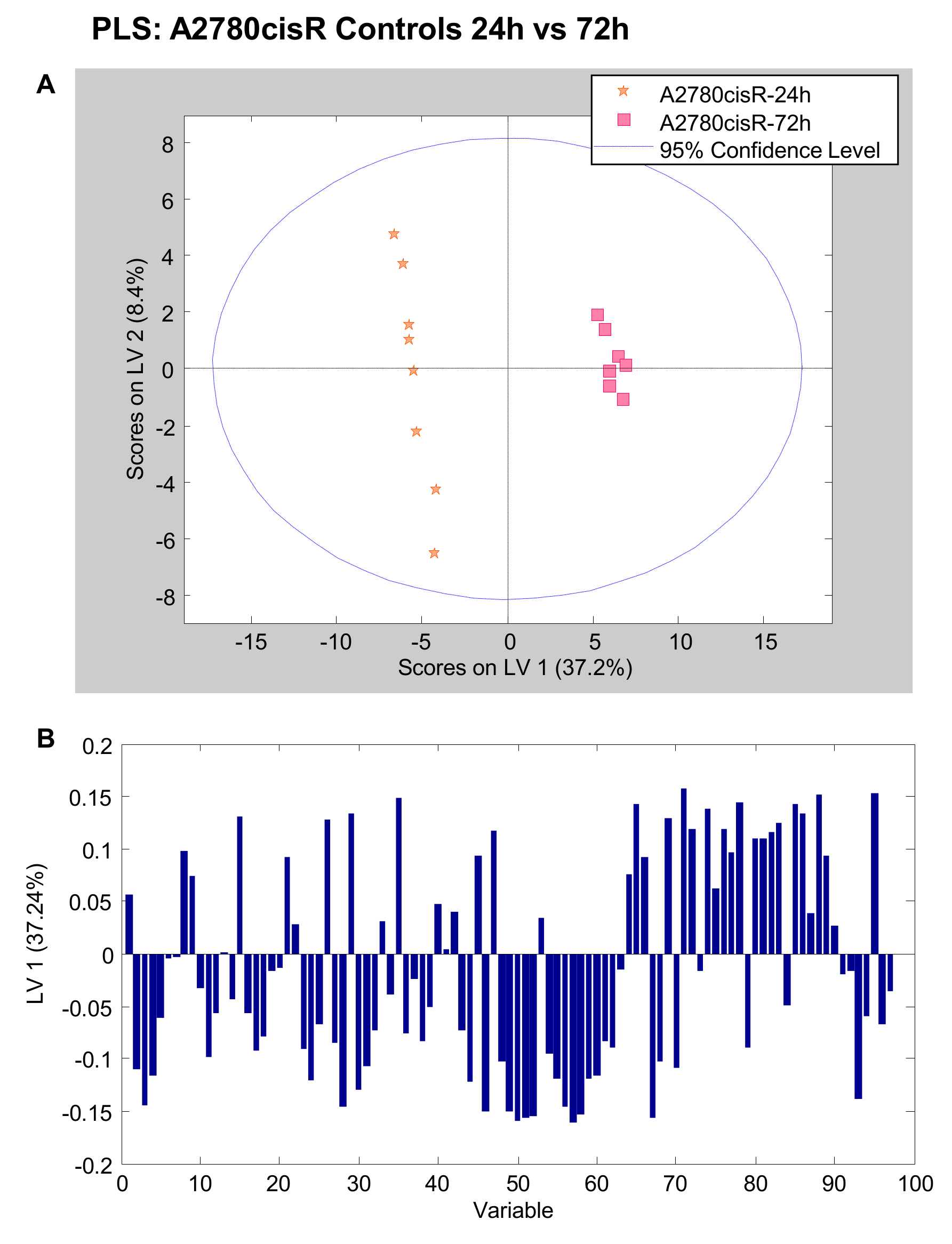

Supplement: S12 Fig — (A) PLS scores plot based on 1D noesy HR-MAS spectral regions between 0.5 and 6.5 ppm (97 buckets) of A2780cisR control cells grown for 24h (red) and 72h (blue). Blue line: 95% confidence level. (B) Corresponding PLS loadings for LV-1. For bucket assignments, see S2 Table. (TIF) [file pone.0128478.s012.tif]

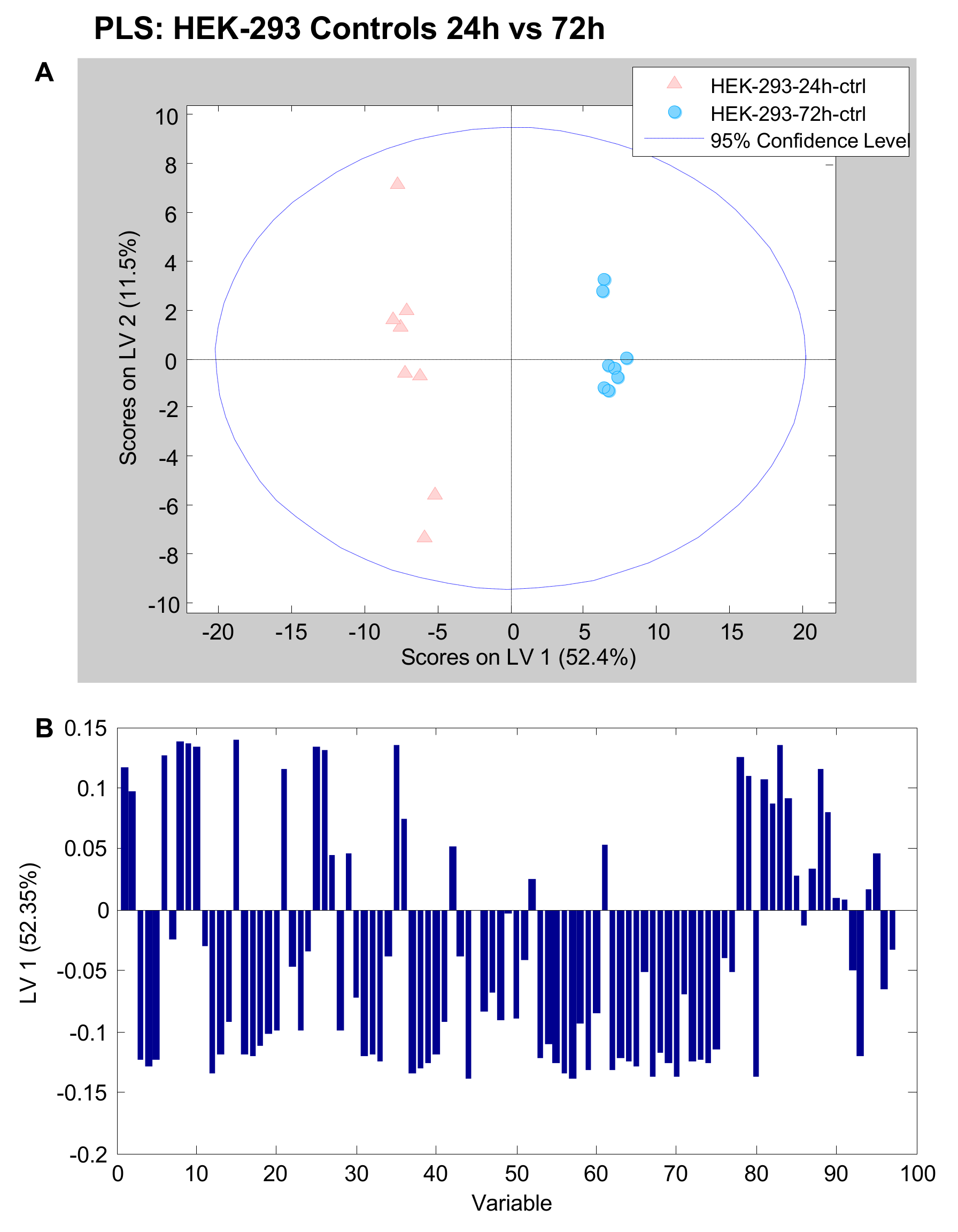

Supplement: S13 Fig — (A) PLS scores plot based on 1D noesy HR-MAS spectral regions between 0.5 and 6.5 ppm (97 buckets) of HEK-293 control cells grown for 24h (red) and 72h (blue). Blue line: 95% confidence level. (B) Corresponding PLS loadings for LV-1. For bucket assignments, see S2 Table. (TIF) [file pone.0128478.s013.tif]

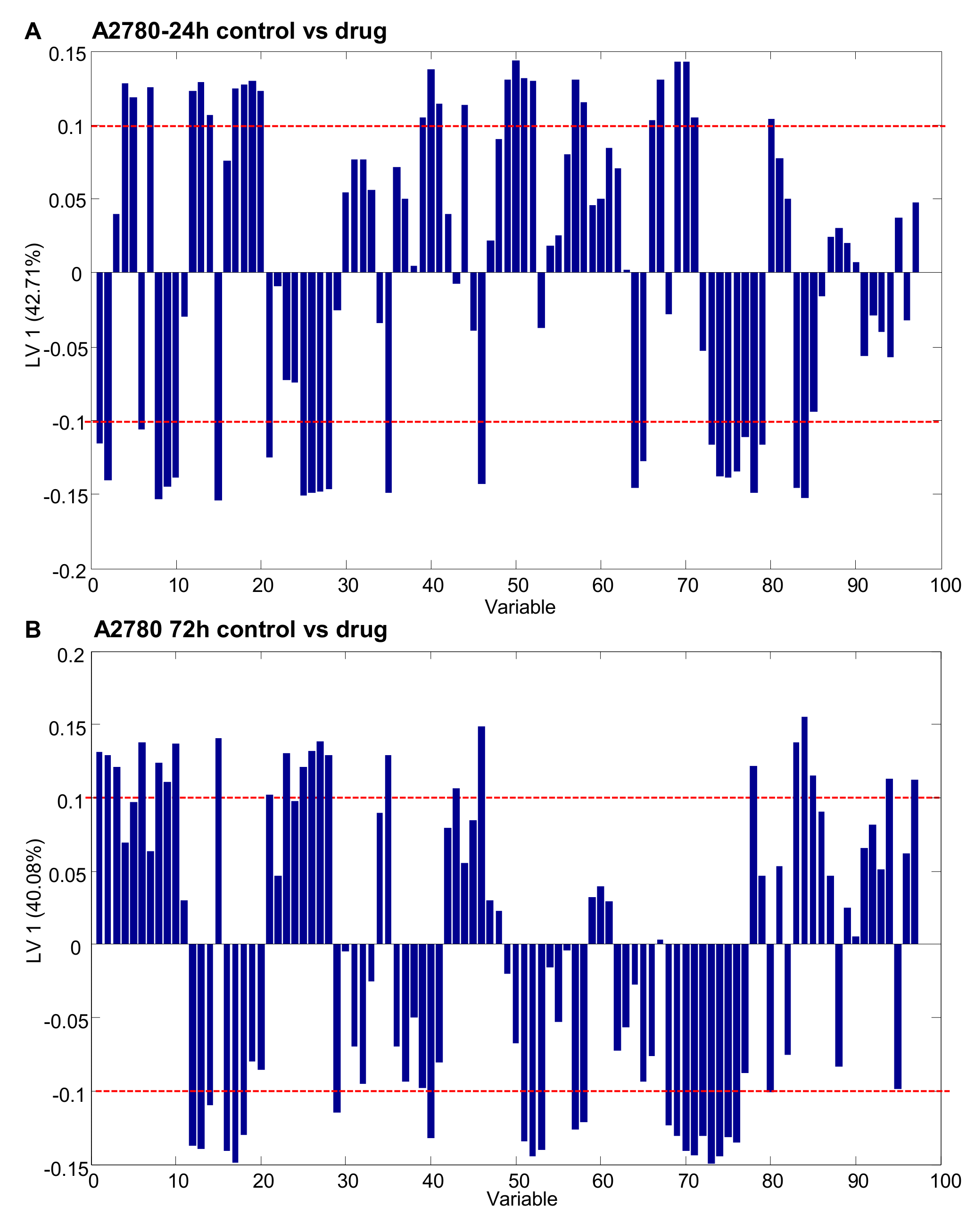

Supplement: S14 Fig — Loading plots for the first PLS component LV1 and 97 variables (= buckets) for PLS comparing A2780 control cells versus drug treated at (A) 24h and (B) 72h. Red line: arbitrary threshold set to a load value of +/- 0.1. (TIF) [file pone.0128478.s014.tif]

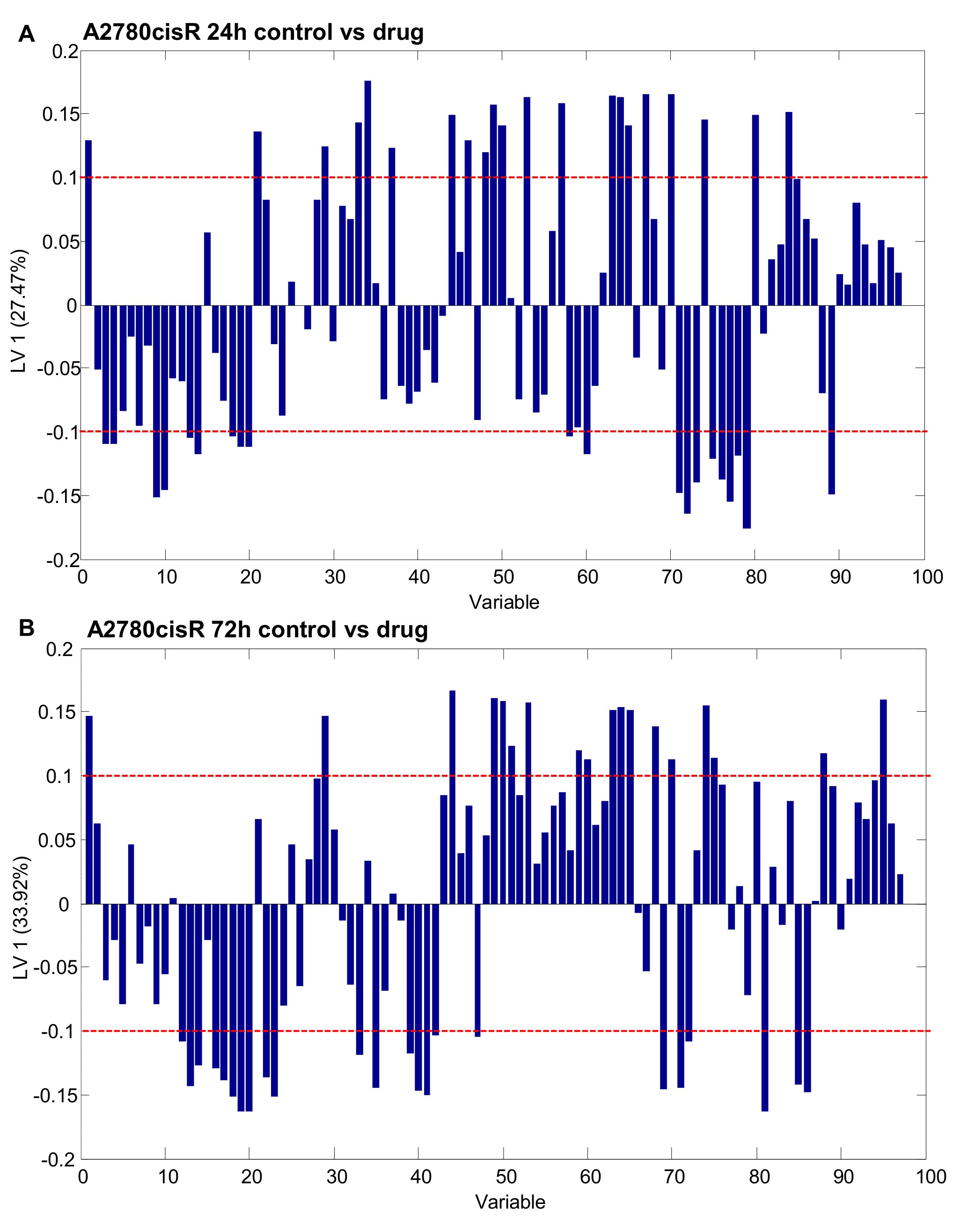

Supplement: S15 Fig — Loading plots for the first PLS component LV1 and 97 variables (= buckets) for PLS comparing A2780cisR control cells versus drug treated at (A) 24h and (B) 72h. Red line: arbitrary threshold set to a load value of +/- 0.1. (TIF) [file pone.0128478.s015.tif]

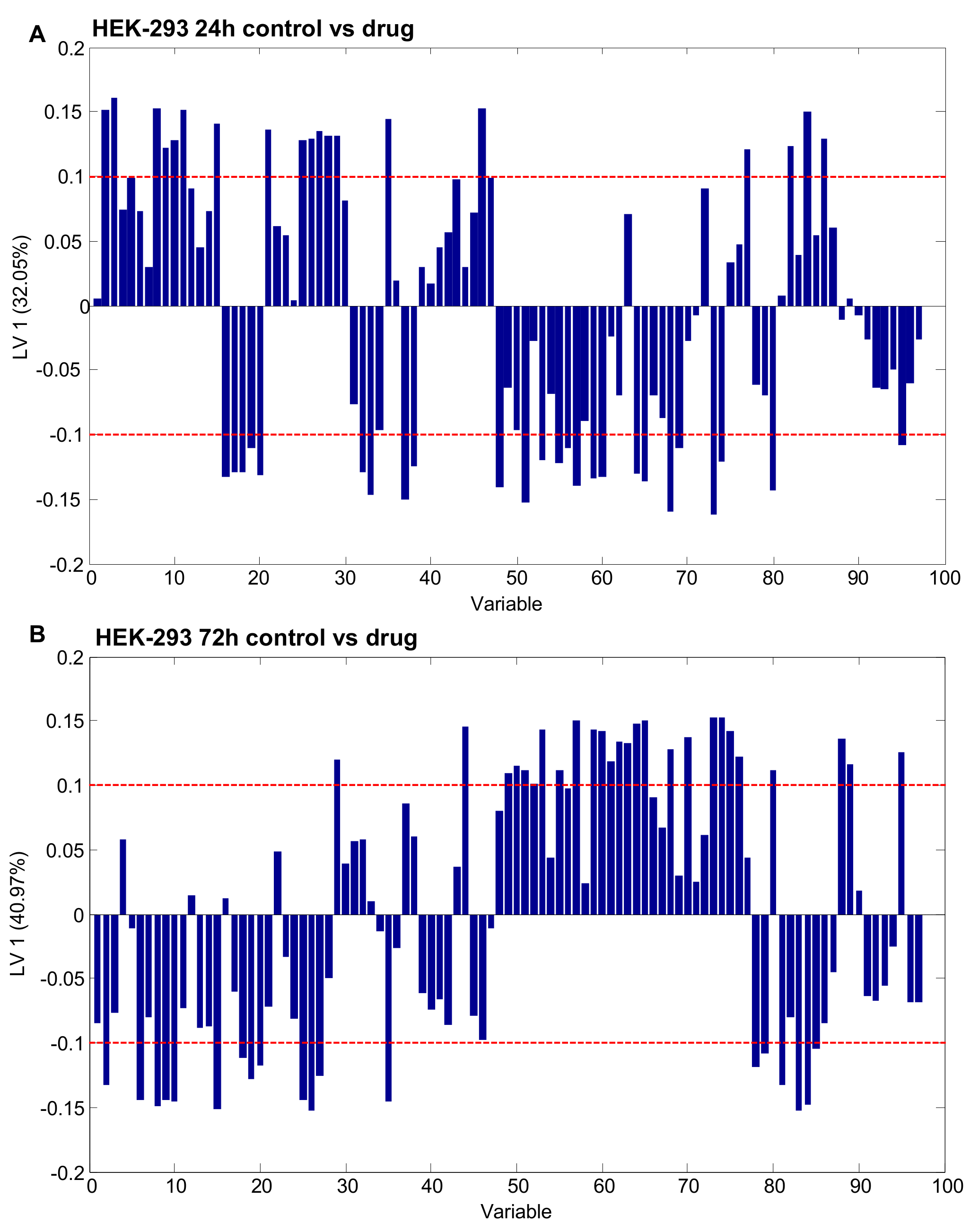

Supplement: S16 Fig — Loading plots for the first PLS component LV1 and 97 variables (= buckets) for PLS comparing HEK-293 control cells versus drug treated at (A) 24h and (B) 72h. Red line: arbitrary threshold set to a load value of +/- 0.1. (TIF) [file pone.0128478.s016.tif]
