# Supplementary material for: 1H HR-MAS NMR Based Metabolic Profiling of Cells in Response to Treatment with a Hexacationic Ruthenium Metallaprism as Potential Anticancer Drug
Source: PLoS One. 2015 May 29;10(5):e0128478. doi: 10.1371/journal.pone.0128478 (PMC4449131; doi:10.1371/journal.pone.0128478)
Supplement: S1 Table — (PDF) [file pone.0128478.s017.pdf]

S1 Table: Normalized mean bucket integrals (± SD) and *p*-values (significant buckets with $p < 0.05$ in red, *p* values corrected for multiple comparisons) for spectral region 5 – 9 ppm.

compound<br>ppm	Glc1P<br>5.458	UNGlc<br>5.51	UNGal<br>5.547	UGlcA<br>5.62	UDP/<br>UTP<br>5.949	Ura<br>5.792	Cyd<br>6.051	Urd<br>5.897	Tyr<br>6.892	Phe<br>7.415	AMP<br>8.21
**A2780 24h**											
ctrl	0.0028<br>±0.0255	0.0511<br>±0.0209	0.0244<br>±0.0183	0.0208<br>±0.0114	0.2392<br>±0.0537	0.0108<br>±0.0186	0.0139<br>±0.0152	0.0591<br>±0.0309	0.0667<br>±0.0336	0.0758<br>±0.0304	0.0311<br>±0.0096
drug	0.0075<br>±0.0183	0.0587<br>±0.0094	0.0296<br>±0.0054	0.0203<br>±0.0135	0.2348<br>±0.0192	0.0060<br>±0.0070	0.0167<br>±0.0111	0.0461<br>±0.0190	0.0585<br>±0.0131	0.0534<br>±0.0134	0.0204<br>±0.0058
p	**7.332**	**4.021**	**5.012**	**10.266**	**9.160**	**5.578**	**7.429**	**3.526**	**5.852**	**0.908**	**0.195**
**A2780 72h**											
ctrl	0.0162<br>±0.0060	0.1108<br>±0.0148	0.0554<br>±0.0060	0.0189<br>±0.0028	0.3025<br>±0.0609	0.0101<br>±0.0031	0.0083<br>±0.0089	0.0355<br>±0.0133	0.0335<br>±0.0083	0.0392<br>±0.0094	0.0202<br>±0.0029
drug	0.0380<br>±0.0174	0.0849<br>±0.0100	0.0435<br>±0.0140	0.0149<br>±0.0096	0.2656<br>±0.0333	0.0096<br>±0.0158	0.0109<br>±0.0129	0.0268<br>±0.0136	0.0608<br>±0.0202	0.0577<br>±0.0121	0.0150<br>±0.0073
P	**0.037**	**0.013**	**0.336**	**2.713**	**1.683**	**10.184**	**6.877**	**2.112**	**0.022**	**0.024**	**0.675**
**A2780cisR 24h**											
ctrl	0.0043<br>±0.0122	0.0360<br>±0.0094	0.0232<br>±0.0102	0.0194<br>±0.0171	0.1267<br>±0.0319	0.0266<br>±0.0206	0.0364<br>±0.0189	0.1022<br>±0.0263	0.0550<br>±0.0070	0.0534<br>±0.0222	0.0306<br>±0.0124
drug	0.0113<br>±0.0130	0.0408<br>±0.0136	0.0155<br>±0.0242	0.0233<br>±0.0169	0.1907<br>±0.0398	0.0349<br>±0.0105	0.0328<br>±0.0138	0.1016<br>±0.0265	0.0490<br>±0.0167	0.0547<br>±0.0106	0.0380<br>±0.0124
P	**3.019**	**4.473**	**4.413**	**7.047**	**0.025**	**3.605**	**7.307**	**10.623**	**3.868**	**9.643**	**2.593**
**A2780cisR 72h**											
ctrl	0.0022<br>±0.0045	0.0991<br>±0.0100	0.0477<br>±0.0063	0.0132<br>±0.0063	0.2607<br>±0.0365	0.0119<br>±0.0044	0.0140<br>±0.0054	0.0298<br>±0.0051	0.0418<br>±0.0133	0.0460<br>±0.0061	0.0220<br>±0.0027
drug	0.0080<br>±0.0083	0.1289<br>±0.0202	0.0668<br>±0.0133	0.0146<br>±0.0148	0.3609<br>±0.0628	0.0131<br>±0.0109	0.0089<br>±0.0076	0.0387<br>±0.0179	0.0230<br>±0.0075	0.0293<br>±0.0160	0.0216<br>±0.0049
P	**0.881**	**0.013**	**0.016**	**8.686**	**0.008**	**8.408**	**1.298**	**1.827**	**0.052**	**0.112**	**8.833**
**HEK-293 24h**											
ctrl	0.0074<br>±0.0076	0.0428<br>±0.0040	0.0247<br>±0.0087	0.0248<br>±0.0060	0.1746<br>±0.0237	0.0278<br>±0.0085	0.0277<br>±0.0083	0.0704<br>±0.0156	0.0311<br>±0.0058	0.0714<br>±0.0113	0.0213<br>±0.0045
drug	0.0128<br>±0.0369	0.0179<br>±0.0287	0.0018<br>±0.0263	0.0091<br>±0.0303	0.1772<br>±0.0547	0.0273<br>±0.0651	0.0111<br>±0.0443	0.0986<br>±0.0692	0.0719<br>±0.0965	-0.0281<br>±0.2279	0.0490<br>±0.0314
P	**3.554**	**8.935**	**9.501**	**6.065**	**9.988**	**10.833**	**4.007**	**3.604**	**3.363**	**3.216**	**0.644**
**HEK-293 72h**											
ctrl	0.0314<br>±0.0167	0.0871<br>±0.0193	0.0376<br>±0.0122	0.0193<br>±0.0100	0.2489<br>±0.0323	0.0094<br>±0.0070	0.0068<br>±0.0122	0.0113<br>±0.0017	0.0234<br>±0.0065	0.0663<br>±0.0115	0.0176<br>±0.0047
drug	0.0200<br>±0.0170	0.1020<br>±0.0080	0.0463<br>±0.0056	0.0179<br>±0.0062	0.3012<br>±0.0434	0.0089<br>±0.0082	0.0057<br>±0.0076	0.0148<br>±0.0047	0.0151<br>±0.0038	0.0474<br>±0.0084	0.0148<br>±0.0060
p	**1.897**	**0.783**	**1.021**	**8.061**	**0.108**	**9.726**	**9.055**	**0.553**	**0.104**	**0.022**	**3.223**
