# Supplementary material for: 1H HR-MAS NMR Based Metabolic Profiling of Cells in Response to Treatment with a Hexacationic Ruthenium Metallaprism as Potential Anticancer Drug
Source: PLoS One. 2015 May 29;10(5):e0128478. doi: 10.1371/journal.pone.0128478 (PMC4449131; doi:10.1371/journal.pone.0128478)
Supplement: S2 Table — (PDF) [file pone.0128478.s018.pdf]

S2 Table: Assignment and spectral regions of buckets.

Bucket number	Compound	Bucket right limit [ppm]	Bucket left limit [ppm]
1	Cholest. esters	0.679	0.759
2	Lip ω-**CH_3-**	0.832	0.931
3	Ile, Leu	0.943	0.982
4		0.991	1.004
5	Ile	1.004	1.021
6	Val	1.021	1.055
7		1.055	1.082
8	Lip -$(CH_2)_n$-	1.253	1.325
9	Lip + Lac	1.325	1.341
10	Lip + Lac	1.341	1.358
11	Lip + (Thre, Lac)	1.380	1.449
12	Lys	1.461	1.480
13	Ala	1.480	1.495
14		1.495	1.514
15	Lip β-**CH_2-**	1.516	1.647
16		1.651	1.709
17	Lys	1.709	1.721
18	Leu	1.721	1.738
19		1.738	1.753
20		1.753	1.769
21		1.815	1.897
22		1.897	1.907
23		1.908	1.920
24	Ac	1.920	1.940
25	Lip **-CH_2-**CH=CH-	1.984	2.037
26	Lip **-CH_2-**CH=CH-	2.037	2.051
27	Lip **-CH_2-**CH=CH-	2.051	2.065
28	Lip **-CH_2-**CH=CH-	2.065	2.081
29	UNGlc, UNGal	2.082	2.103
30		2.106	2.119
31		2.119	2.134
32		2.134	2.148
33		2.148	2.163
34	Glu/GSH	2.163	2.216
35	Lip α-**CH_2-**	2.220	2.280
36	Val	2.286	2.332
37	Gln	2.341	2.376

Bucket number	Compound	Bucket right limit [ppm]	Bucket left limit [ppm]
38		2.376	2.394
39		2.394	2.406
40		2.406	2.423
41		2.423	2.436
42		2.492	2.511
43		2.526	2.543
44	Glu/GSH	2.545	2.596
45		2.653	2.685
46	Lip =CH-**CH_2**-CH=	2.782	2.871
47		2.871	2.890
48		2.907	2.941
49	GSH	2.941	2.971
50		2.971	2.996
51	Lys	2.998	3.028
52		3.028	3.039
53	Cre	3.039	3.059
54		3.147	3.174
55		3.174	3.194
56	Cho	3.194	3.219
57	PC (PE)	3.219	3.246
58	GPC	3.246	3.291
59	m-Ino	3.291	3.306
60		3.307	3.324
61		3.336	3.357
62		3.357	3.378
63		3.425	3.467
64	Cho	3.525	3.547
65	m-Ino	3.547	3.570
66		3.570	3.589
67	PC, Thre	3.589	3.617
68		3.617	3.630
69	Glu/GSH, Ala, Arg	3.749	3.780
70	Glu/GSH	3.780	3.813
71		3.814	3.852
72		3.852	3.888
73		3.888	3.929
74	Cre	3.929	3.949
75	Tyr	3.950	3.976
76		3.976	4.003
77	PE	4.004	4.039

Bucket number	Compound	Bucket right limit [ppm]	Bucket left limit [ppm]
78	Cho	4.048	4.095
79	Lac	4.095	4.154
80	PC	4.156	4.228
81		4.991	5.026
82		5.110	5.173
83	Glc or glycogen	5.180	5.236
84	Lip -CH=CH-	5.260	5.399
85	GlcN	5.414	5.439
86		5.439	5.460
87	Glc1P	5.460	5.500
88	UNGlc	5.511	5.549
89	UNGal	5.549	5.585
90	UGlcA	5.617	5.667
91		5.738	5.765
92	Ura	5.801	5.835
93	Urd	5.902	5.937
94		5.937	5.959
95	UDP/UTP	5.959	6.032
96	Cyd	6.054	6.098
97		6.109	6.192
